# Assessment of predictive performance in incomplete data by combining internal validation and multiple imputation

**DOI:** 10.1186/s12874-016-0239-7

**Published:** 2016-10-26

**Authors:** Simone Wahl, Anne-Laure Boulesteix, Astrid Zierer, Barbara Thorand, Mark Avan de Wiel

**Affiliations:** 1 Research Unit of Molecular Epidemiology, Helmholtz Zentrum München - German Research Center for Environmental Health, Ingolstädter Landstrasse, Neuherberg, 1, 85764 Germany; 2 Institute of Epidemiology II, Helmholtz Zentrum München - German Research Center for Environmental Health, Ingolstädter Landstrasse, Neuherberg, 1, 85764 Germany; 3 German Center for Diabetes Research (DZD e.V.), Ingolstädter Landstrasse, Neuherberg, 1, 85764 Germany; 4 Department of Medical Informatics, Biometry and Epidemiology, Ludwig-Maximilians-Universität München, Marchioninistrasse, Munich, 15, 81377 Germany; 5 Department of Epidemiology and Biostatistics, VU University Medical Center, PO Box 7057, Amsterdam, 1007 MB The Netherlands; 6 Department of Mathematics, VU University, De Boelelaan 1081a, Amsterdam, 1081 HV The Netherlands

**Keywords:** Missing values, Incomplete data, Prediction model, Predictive performance, Bootstrap, Internal validation, Resampling, Cross-validation, Multiple imputation, MICE

## Abstract

**Background:**

Missing values are a frequent issue in human studies. In many situations, multiple imputation (MI) is an appropriate missing data handling strategy, whereby missing values are imputed multiple times, the analysis is performed in every imputed data set, and the obtained estimates are pooled. If the aim is to estimate (added) predictive performance measures, such as (change in) the area under the receiver-operating characteristic curve (AUC), internal validation strategies become desirable in order to correct for optimism. It is not fully understood how internal validation should be combined with multiple imputation.

**Methods:**

In a comprehensive simulation study and in a real data set based on blood markers as predictors for mortality, we compare three combination strategies: *Val-MI*, internal validation followed by MI on the training and test parts separately, *MI-Val*, MI on the full data set followed by internal validation, and *MI(-y)-Val*, MI on the full data set omitting the outcome followed by internal validation. Different validation strategies, including bootstrap und cross-validation, different (added) performance measures, and various data characteristics are considered, and the strategies are evaluated with regard to bias and mean squared error of the obtained performance estimates. In addition, we elaborate on the number of resamples and imputations to be used, and adopt a strategy for confidence interval construction to incomplete data.

**Results:**

Internal validation is essential in order to avoid optimism, with the bootstrap 0.632+ estimate representing a reliable method to correct for optimism. While estimates obtained by *MI-Val* are optimistically biased, those obtained by *MI(-y)-Val* tend to be pessimistic in the presence of a true underlying effect. *Val-MI* provides largely unbiased estimates, with a slight pessimistic bias with increasing true effect size, number of covariates and decreasing sample size. In *Val-MI*, accuracy of the estimate is more strongly improved by increasing the number of bootstrap draws rather than the number of imputations. With a simple integrated approach, valid confidence intervals for performance estimates can be obtained.

**Conclusions:**

When prognostic models are developed on incomplete data, *Val-MI* represents a valid strategy to obtain estimates of predictive performance measures.

**Electronic supplementary material:**

The online version of this article (doi:10.1186/s12874-016-0239-7) contains supplementary material, which is available to authorized users.

## Background

The aim of a prognostic study is to develop a classification model from an available data set and to estimate the performance it would have in future independent data, i.e., its *predictive* performance. This cannot be achieved by fitting the model on the whole data set and evaluating performance in the same data set, since a model generally performs better for the data used to fit the model than for new data (“overfitting”) and performance would thus be overestimated. This can be observed already in low-dimensional situations and is especially pronounced in relatively small data sets [1, 2]. Instead, the available data have to be split in order to allow performance assessment in a part of the data that has not been involved in model fitting [3, 4]. For efficient sample usage, this is often achieved by internal validation strategies such as bootstrapping (BS), subsampling (SS) or cross-validation (CV).

The task of assessing predictive performance is made even more complicated when the data set is incomplete. Missing values occur frequently in epidemiological and clinical studies, for reasons such as incomplete questionnaire response, lack of biological samples, or resource-based selection of samples for expensive laboratory measurements. The majority of statistical methods, including logistic regression models, assume a complete data matrix, so that some action is required prior to or during data analysis to allow usage of incomplete data. Since ad hoc strategies such as complete-case analysis and single imputation often provide inefficient or invalid results, and model-based strategies require often sophisticated problem-specific implementation, multiple imputation (MI) is becoming increasingly popular among researchers of different fields [5, 6]. It is a flexible strategy that typically assumes *missing at random* (MAR) missingness, that is, missingness depending on observed but not unobserved data, which is often, at least approximately, given in practice [5]. MI involves three steps [7]: (i) missing values are imputed multiple (*M*) times, i.e., missing values are replaced by plausible values, for instance derived as predicted values from a sequence of regression models including other variables, (ii) statistical analysis is performed on each of the resulting completed data sets, and (iii) the *M* obtained parameter estimates and their variances are pooled, taking into account the uncertainty about the imputed values [8].

When the estimate of interest is a measure of predictive performance of a classification model, or a measure of incremental predictive performance of an extended model as compared to a baseline model, the application of MI is not straightforward. Specifically, it is unclear how internal validation and MI should be combined in order to obtain unbiased estimates of predictive performance.

Previous strategies combining internal validation with MI mostly focused on application without the aim to compare their chosen strategy against others or to assess their validity [9–11]. Musoro et al. [12] studied the combination of BS and MI in the situation of a nearly continuous outcome using LASSO regression, essentially reporting that the strategy of conducting MI first followed by BS on the imputed data yielded overoptimistic mean squared errors, whereas conducting BS first on the incomplete data followed by MI yielded slightly pessimistic results in the studied settings. Wood et al. [13] presented a number of strategies for performance assessment in multiply imputed data, leaving, however, the necessity of validating the model in independent data to future studies. Hornung et al. [14] examined the consequence of conducting a single imputation on the whole data set as compared to the training data set on cross-validated performance of classification methods, observing a negligible influence. Their investigation was restricted to one type of imputation that did not include the outcome in the imputation process.

In this paper, we present results of a comprehensive simulation study and results of a real data-based simulation study comparing various strategies of combining internal validation with MI, with and without including the outcome in the imputation models. Our study extends upon previous work with regard to several aspects: (1) We consider different internal validation strategies and different ways to correct for optimism, we (2) study measures of discrimination, calibration and overall performance as well as incremental performance of an extended model, and we (3) closely examine the sensitivity of the results towards characteristics of the data set, including sample size, number of covariates, true effect size and degree and mechanism of missingness. Furthermore, we (4) elaborate on the number of imputations and resamples to be used and (5) provide an approach for the construction of confidence intervals for predictive performance estimates. Finally, we (6) translate our results into recommendations for practice, considering the applicability of the proposed methods for epidemiologists with limited analytical and computational resources.

## Methods

### Study data

Two simulation studies were conducted: In the first, incomplete data were generated *de novo* with different (known) effect sizes, facilitating the comparison of predictive performance estimates of different combined validation/imputation strategies against the respective true performance measure. The second simulation study was based on the complete observations of a real incomplete data set, in which we introduced missing values in a pattern mirroring that of the whole incomplete data set, aiming to compare strategies in a realistic data situation.

#### Simulation study 1: de novo simulation

##### Data generation

Data were generated according to a variety of settings, covering a large spectrum of practically occurring data characteristics (Table 1). For each setting, 250 data sets were randomly generated. Two situations were investigated. In situation 1, only one set of covariates was considered (the number of which is denoted as *p*), with the aim being the estimation of predictive performance of a model comprising this set of covariates. In situation 2, two sets of covariates were considered (with *p*_0_ the number of baseline covariates and *p*_1_ the number of additional covariates), in order to study the estimation of added predictive performance of the model comprising both sets of covariates as compared to a model containing only the *p*_0_ baseline covariates.

**Table 1:** Simulation settings

Parameter	Notation	Values	
*Predictive performance*			
Sample size	*n*	100, 200, 500, 1000	
Number of covariates	*p*	1, 5, 10, 20	
Correlation among covariates	*ρ*	0, 0.25	
Outcome case frequency	*frac*	0.5, 0.25	
Theoretical AUC	*auc*	0.5, 0.58, 0.66, 0.74, 0.82	
Proportion of missing values among covariates	*miss*	0.125, 0.25, 0.375, 0.5, 0.625, 0.75	
Missingness mechanism		MCAR, MAR, MARblock	
*Added predictive performance*			
		Baseline covariates	Additional covariates
Sample size	*n*	100, 200, 500, 1000	
Number of covariates	*p*_0_, *p*_1_	1, 5, 10	1,5,10,20
Correlation among covariates	*ρ*_0_, *ρ*_1_	0	0, 0.25
Outcome case frequency	*frac*	0.5, 0.25	
Theoretical (change in) AUC	*auc*_0_, *Δ* *auc*	0.6	0, 0.04, 0.08, 0.12, 0.16
Proportion of missing values among covariates	*miss*_0_, *miss*_1_	0, 0.5	0.125, 0.25, 0.375, 0.5, 0.625, 0.75
Missingness mechanism		MCAR, MAR, MARblock	

*AUC* area under the receiver-operating characteristic (ROC) curve, *MAR* missing at random, *MARblock* blockwise missing at random, *MCAR* missing completely at random

For each simulated data set, a binary outcome vector ***y***=(*y*_1_,*y*_2_,…,*y*_*n*_) was created with the pre-specified case probability *frac*. A covariate matrix **X**=(***x***_1_,***x***_2_,…,***x***_*n*_) was simulated by drawing *n* times from a *p* or *p*_0_+*p*_1_-dimensional (in situations 1 and 2, respectively) multivariate normal distribution with mean vector ***0*** and variance-covariance matrix **Σ** with variances equal to 1 and covariances specified by the correlation among variables (*ρ* in situation 1, *ρ*_0_ and *ρ*_1_ for the baseline and additional covariates, respectively, in situation 2) as provided in Table 1. Then, effect sizes were introduced in a way that each set of covariates achieved an (added) performance approximately in the magnitude of a pre-specified area under the receiver-operating characteristic (ROC) curve (AUC) value. As a reference we used the theoretical relationship [15]:

1 $$ \text{AUC} = \Phi\left(\frac{1}{2} \sqrt{\boldsymbol{\Delta\mu}^{T} \mathbf{\Sigma^{-1}} \boldsymbol{\Delta\mu}} \right),  $$

where ***Δμ*** denotes the vector of mean differences in covariate values to be introduced between both outcome classes, i.e., ***Δμ***=*E*(***x***_*i*_|*y*_*i*_=1)−*E*(***x***_*i*_|*y*_*i*_=0), and *Φ* the standard normal cumulative distribution function. We used a simplified scenario with a unique effect size chosen for all covariates within each set, i.e., ***Δμ***=(*Δ* *μ*,*Δ* *μ*,…,*Δ* *μ*) in situation 1, and ***Δμ***=(*Δ* *μ*_0_,*Δ* *μ*_0_,…,*Δ* *μ*_0_,…,*Δ* *μ*_1_,*Δ* *μ*_1_,…,*Δ* *μ*_1_) in situation 2, and found ***Δμ*** by solving Eq. (1) numerically using the R function *uniroot*. Then, we added ***Δμ***/2 to the cases’ covariate values, and substracted ***Δμ***/2 from the controls’ covariate values, in order to achieve an average difference of ***Δμ*** in covariate values between cases and controls. Using this procedure, we implicitly model the outcome *y*_*i*_ as follows: *P*(*y*_*i*_=1|***x***_*i*_)=logistic(*γ*·***x***_*i*_), where ***x***_*i*_ denotes the vector of covariate values for observation *i*, *i*=1,…,*n*, *γ*=**Σ** ^−1^·***Δμ*** a *p*-dimensional (situation 1) or *p*_0_+*p*_1_-dimensional (situation 2) vector of coefficients, and $\text {logistic}(x) = \frac {e^{x}}{1+e^{x}}$ the logistic function.

##### Imposing missingness

Different degrees of missingness (see Table 1) were introduced separately to the sets of covariates (one set in situation 1 with proportion of missing values denoted as *miss*; two sets in situation 2 with proportion of missing values in the baseline and additional covariates denoted as *miss*_0_ and *miss*_1_, respectively; to improve readability, we use the parameter notations of situation 1 below) according to three different mechanisms frequently occurring in practice: missing completely at random (MCAR), where missingness occurs independently of any observed or missing values, missing at random (MAR), where missingness of variables depends on observed values including outcome values but not on the unknown values of the missing data, and blockwise missing at random (MARblock), where blocks of variables share their missingness pattern. We did not consider missingness in the outcome.

MCAR missingness was created by randomly introducing the pre-specified proportion *miss* of missing values into the covariates. To achieve MAR missingness, we used an approach similar to that applied by Marshall et al. [16]. Let *X*_*ij*_ denote the *j* ^th^ covariate for observation *i*, with *i*=1,…,*n* and *j*=1,…,*p*, and *M*_*ij*_ the indicator for its missingness. Then, the probability of missingness for each covariate value was modeled as a function of the value of one other covariate, of missingness of another covariate, and of the outcome value.

$$\begin{array}{*{20}l} & P\left(X_{\mathit{ij}}~\text{missing}\right) = P\left(M_{\mathit{ij}} = 1\right)\\ & = \text{logistic}\left(\beta_{0j} + \beta_{1j} \cdot M_{i,j-1} + \beta_{2j} \cdot X_{ik_{j}} + 2 \cdot y_{i}\right) \end{array} $$

where $X_{ik_{j}}$ denotes the observation of a randomly chosen other covariate and *y*_*i*_ the binary outcome value. Without loss of generality, missingness of the previous (*j*−1th) covariate was used for technical reasons (missingness available). *β*_1*j*_ was defined as

$$\begin{array}{*{20}l} \beta_{1j} = \left\{ \begin{array}{ll} 0,& \quad\text{if}~j=1\\ 1;-1~\text{with probability}~0.5, & \quad\text{if}~j>1\\ \end{array}\right. \end{array} $$

and *β*_2*j*_ as

$$\begin{array}{*{20}l} \beta_{2j} = \left\{ \begin{array}{ll} 0,& \quad\text{if }j=0\\ 2, & \quad\text{if }j>1.\\ \end{array}\right. \end{array} $$

The intercepts *β*_0*j*_ were estimated by numerically solving the equation

$$\frac{1}{n}\sum_{i=1}^{n} P\left(M_{\mathit{ij}} = 1\right) = \mathit{miss} $$

for each *j*. To achieve the proportion of missing values *miss* exactly, values were set to missing by drawing *n*×*miss* times from a multinomial distribution with probability vector (*P*(*M*_*ij*_=1))_*i*=1,…,*n*_.

Finally, we created a missingness structure similar to that observed in our application data, that is, a block structure of missingness (MARblock). In practice, such a structure can occur when groups of laboratory parameters are measured for certain groups of subjects defined by other variables (see below). Approximate blockwise missingness was simulated with missingness of variables assigned to each block depending on covariates outside the block. Variables were randomly assigned to three blocks, and probability of missingness modified as follows for the covariates *j* in each block *b*, *b*=1,…,3:

$$\begin{array}{*{20}l} & P\left(M_{\mathit{ij}} = 1\right) = \text{logistic}\left(\beta_{0j} + 10 \cdot X_{ik_{b}} + 2 \cdot y_{i}\right) \end{array} $$

where for each covariate *j* within block *b* the same covariate $X_{k_{b}}$ was chosen among covariates outside the block, leading to similarly high/low probabilities for all covariates in the respective block. The exact proportion of missing values *miss* was again achieved by drawing from a multinomial distribution, as described for MAR above. Example R code for simulation study 1 is available in Additional file 2.

#### Simulation study 2: real data-based simulation

##### Data set

Data were obtained from the population-based research platform MONICA (MONItoring of trends and determinants in CArdiovascular disease)/ KORA (COoperative health research in the Region of Augsburg), surveys S1 (1984/85), S2 (1989/90) and S3 (1994/95), comprising individuals of German nationality aged 25 to 74 years. The study design and data collection have been described in detail elsewhere [17]. Written informed consent was obtained from all participants and the studies were approved by the local ethics committee.

In a random subcohort comprising 2225 participants aged 35 to 74 years, blood concentrations of 15 inflammatory markers were measured [18–20] as part of a case-cohort study assessing potential risk factors for cardiovascular diseases and type 2 diabetes. In the present analysis, all-cause mortality was used as the outcome. To achieve a largely healthy population at baseline, subjects with a history of stroke, myocardial infarction, cancer or diabetes at baseline were excluded. Among the remaining 2012 subjects, 294 (14.6 %) died during the 15-year follow-up period. Average survival time among the deceased participants was 9.0 years (range 0.2 to 15.0 years), and three participants were censored at 2.7, 6.9 and 7.9 years. See Additional file 1: Table S1 for a description of baseline phenotypes including the inflammatory markers.

**Fig. 1:**
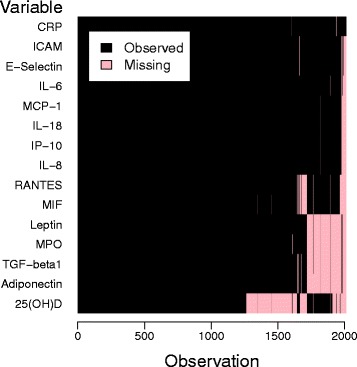
Missingness pattern among inflammation-related markers in the application data set. Plot of missingness indicators (*black* = entry observed; *red* = entry missing) for the 2012 observations against the 15 inflammation-related markers, both sorted by missingness

Whereas all other variables were almost completely observed (less than 0.4 % missing entries for each variable), missingness among the 15 inflammation-related markers was 7.2 % on average (range 0.2 – 26.4 %, see Additional file 1: Table S1), 37.2 % of observations had missing entries in inflammation-related markers, with missingness ranging from 0 to 93.3 %. The missingness pattern showed a block structure (Fig. 1), owing to the fact that measurement of inflammatory markers was conducted in different laboratory runs – for which samples were selected based on sample availability at the time of measurement. Five blocks of covariates could be roughly distinguished: Block 1, comprising CRP, without missings, block 2, conprising ICAM, E-Selectin, IL-6, MCP-1, IL-18,IP-10 and IL-8, block 3, comprising RANTES and MIF, block 4, comprising leptin, MPO, TGF- *β*1 and Adiponectin, and block 5, comprising 25(OH)D. Similarly, observations could be assigned to five patterns of missingness: pattern 1, comprising observations with a missing entry only for block 2, 3, 4 and 5 variables, pattern 2, only for block 4 and 5 variables, pattern 3, only for block 4 variables, pattern 4, only for block 3 and 5 variables, and pattern 5, only for the block 5 variable 25(OH)D.

##### Imposing missingness

To use the MONICA/KORA subcohort as the basis for the real data-based simulation study, we first investigated determinants of missingness in inflammation-related markers in the full subcohort, followed by imposing missingness on the data set consisting of the complete observations only (*n*=1258) in a way that yielded a missingness pattern closely resembling the block structure and the relations in the original data set. In detail, we used the five patterns of missingness described above as a basis, and, for each pattern, identified other variables in the data set correlated (Kendall’s *τ*) with the respective pattern indicator (1 for observations that are part of the respective pattern; 0 else). Consequently, we selected those variables showing an absolute correlation above 0.1: sex and survey 1 for pattern 1, survey 1 for pattern 2, sex, survey 1 and alcohol intake for pattern 3, and no covariates for pattern 4 and 5. 250 simulations were conducted. In each simulation, a proportion of complete observations was assigned to each pattern identical to the proportion observed in the original data set. This was achieved by modeling pattern indicators as a function of the respective correlated variable(s) in the full incomplete data set in a logistic regression model, and predicting pattern membership probability of the respective pattern for the observations in the complete-observation data set. To achieve the aspired proportion of observations newly assigned to each pattern exactly, we drew the required number of times from a multinomial distribution with the predicted probability vector. Finally, for observations assigned to pattern 1, all variables of blocks 2, 3, 4 and 5 were set to missing, for pattern 2, variables of blocks 4 and 5, and so on, according to the definitions above. The resulting data sets showed a missingness pattern closely resembling that of the original data set (shown for the first 12 simulation runs in Additional file 1: Figure S1).

### Imputation

We used the *multiple imputation by chained equations* (MICE) framework [7, 21]. It is based on the principle of a repeated chain of regression equations through the incomplete variables, where in each imputation model, the respective incomplete variable is modeled as a function of the remaining variables. Arbitrary regression models can be used. We applied *predictive mean matching* for all incomplete (continuous) variables. It is based on Bayesian linear regression, where after modeling, the posterior predictive distribution of the data is specified and used to draw predicted values [22]. Then, missing values are replaced by a random draw of observed values of that variable from other observations with the closest predicted values (default: the five closest values). In each imputation model, all other variables (and, in the data-based simulation study, quadratic terms of continuous variables, passively imputed themselves) were included as covariates. Before imputation, to improve normality of the continuous incomplete variables, distributions of raw, natural logarithm, cubic root and square root transformed variables were tested for normality using Shapiro-Wilk tests, and the transformation yielding the maximum test statistic was applied.

Depending on the strategy used (see below), the outcome was included (strategy *MI*) or not included (strategy *MI(-y)*) in the imputation models. If MI was not combined with internal validation, a pooled performance estimate was obtained by averaging the performance estimates $\hat \theta ^{(m)}$, *m*=1,…,*M*, from the *M* imputed data sets, according to Rubin [8]. Example R code for the conduction of MICE is available in Additional file 2.

### Internal validation strategies

**Fig. 2:**
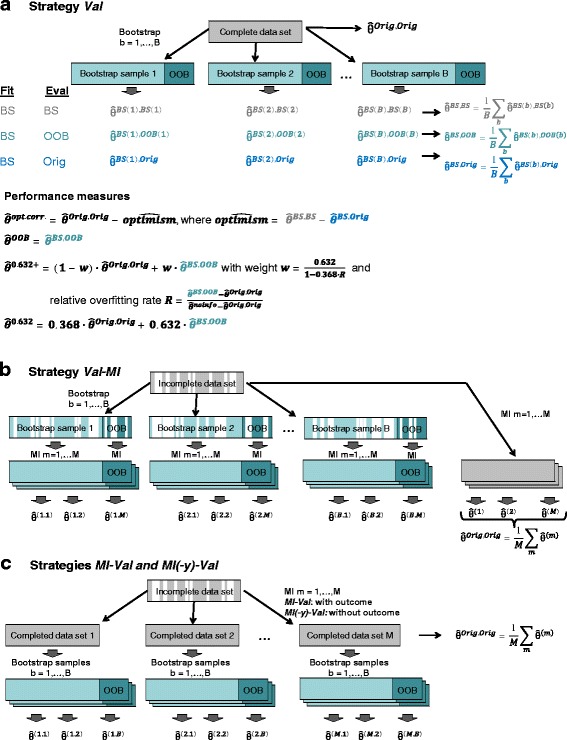
Combination of internal validation (*Val*), using the example of bootstrap (*BS*), and multiple imputation (*MI*). **a** *Val*: Visualization of BS in complete data. $\hat \theta ^{{Dat}_{1},{Dat}_{2}}$ denotes performance when the model was fitted on *Dat*_1_ and evaluated on *Dat*_2_, where *Orig* denotes the original data set, *BS(b)* the *b*th BS set, *OOB(b)* the *b*th out-of-bag (OOB) set, *b*=1,…,*B*. Average performance values across the *B* sets are denoted by $\hat \theta ^{{BS,BS}}$, $\hat \theta ^{{BS, OOB}}$ and $\hat \theta ^{{BS, Orig}}$. $\hat \theta ^{{noinfo}}$ denotes the average performance in the absence of an effect (see text). *Performance measures*: $\hat \theta ^{{opt.corr.}}$, ordinary optimism-corrected BS estimate [3]; $\hat \theta ^{{OOB}}$, OOB performance estimate; $\hat \theta ^{0.632+}$, BS 0.632+ estimate [23]. In the specific case of *w*=0.632, the BS 0.632 estimate ($\hat \theta ^{0.632}$) is obtained. **b** *Val-MI*: Combination of *BS* and *MI* by drawing BS samples followed by MI separately on the BS samples and on the OOB samples not contained in the respective BS draw. **c** *MI-Val* and *MI(-y)-Val*: Combination of *MI* and *BS* by conducting MI followed by drawing BS samples from the imputed data sets. For **b** and **c**, performance measures are derived similarly as for complete data (**a**), this time averaging across the *B*·*M* sets, and deriving apparent performance $\hat \theta ^{{Orig,Orig}}$ as the average performance across the *M* imputed data sets

Three internal validation strategies were considered: bootstrapping (BS), subsampling (SS) and *K*-fold cross-validation (CV). The principles underlying the three strategies are visualized for complete data in Fig. 2 a (BS) and in Additional file 1: Figure S2 (SS, CV).

Briefly, in BS, *B* bootstrap samples are drawn with replacement from the original sample, so that each BS sample will contain certain observations more than once, and others not at all. The average proportion of independent observations included in each BS sample is asymptotically 63.2 % [23]. The approx. 36.8 % remaining observations are frequently referred to as the *out-of-bag* (OOB) sample. To get an estimate for predictive performance from BS, several strategies were proposed (Fig. 2). First, the *optimism* of the apparent performance $\hat \theta ^{\text {Orig,Orig}}$ (i.e., the performance of the model in the original data after using the whole original data set for model fitting), can be estimated as difference between average apparent performance in the BS samples and average performance of models fitted in each BS sample evaluated in the original sample [3]: $\widehat {{optimism}} = \hat \theta ^{{BS,BS}} - \hat \theta ^{{BS,Orig}}$. Accordingly, an “optimism-corrected” (*opt.corr.*) measure for predictive performance, sometimes referred to as ordinary bootstrap estimate, can be obtained by subtracting the estimated optimism from apparent performance in the original data: $\hat \theta ^{{opt.corr.}} = \hat \theta ^{{Orig,Orig}} - \widehat {{optimism}}$. Second, the model can be fitted on the BS samples and evaluated on the OOB samples ($\hat \theta ^{\text {OOB}}$). The resulting performance estimate tends to underestimate performance since less information was used in the model fitting step than provided in the full data [24]. Thus, the BS 0.632+ estimate ($\hat \theta ^{\text {0.632+}}$) has been proposed as a weighted average of apparent and OOB performance:

$$\hat\theta^{\text{0.632+}} = (1-w) \cdot \hat\theta^{\text{\textit{Orig,Orig}}} + w \cdot \hat\theta^{\text{OOB}} $$

with weights $w = \frac {0.632}{1-0.368 \cdot R}$ depending on the relative overfitting rate $R=\frac {\hat \theta ^{{OOB}}-\hat \theta ^{{Orig,Orig}}}{\hat \theta ^{{noinfo}}-\hat \theta ^{{Orig,Orig}}}$ (Fig. 2, [23]). This requires that we know the performance of the model in the absence of an effect ($\hat \theta ^{\text {noinfo}}$), which is either known (e.g., 0.5 in the case of the AUC, and 0 in the case of added predictive performance measures) or can be approximated as the average performance measure with randomly permuted outcome prediction. We used 1000 permutations to assess $\hat \theta ^{\text {noinfo}}$ for the Brier score. In addition, we considered the BS 0.632 estimate $\hat \theta ^{\text {0.632}} = 0.368 \cdot \hat \theta ^{{Orig,Orig}} + 0.632 \cdot \hat \theta ^{\text {OOB}}$ [25].

SS and CV involve drawing without replacement. For SS, we sampled a proportion 63.2 % of samples for model fitting, leaving again 36.8 % for evaluation. The optimism correction methods described for the BS can be directly translated to SS. For *K*-fold CV, the sample is split in *K* equally sized parts, and for each of the parts, the remaining *K*−1 parts are used for model fitting and the left-out part for evaluation of the model, followed by averaging the performance estimates obtained from the *K* runs. We used *K*=3 and *K*=10, with the former being comparable to BS in terms of the proportion of independent observations in the training sets, and the latter being a popular choice in the literature. Repeating *K*-fold CV *B* times and averaging the resulting performance estimates might improve stability of performance evaluation [2]. Thus, both simple (*CV3*, *CV10*) and repeated (*CV3rep*, *CV10rep*) CV with *K*=3 and *K*=10, respectively, were included in the investigation.

### Combination of internal validation with multiple imputation

Simulated and real incomplete data were analyzed according to three combination strategies: Internal validation data splits followed by MI of the training/fitting and testing/evaluation data parts separately (*Val-MI*), and performing the internal validation on multiply imputed data with (*MI-Val*) and without (*MI(-y)-Val*) having included the outcome in the imputation models. Thereby *Val* represents the different validation strategies used, i.e., *BS*, *SS*, *CVK* and *CVKrep*. A visualization is provided for BS in Fig. 2. When performing MI, it is generally recommended to use the outcome data *y* in the imputation models for missing covariates (i.e., method *MI-Val*) [26]. However, in the present context, where we split the imputed data into a training and an evaluation set (*Val*), we may want to consider removing *y* from the imputation models (i.e., method *MI(-y)-Val*) because these models are fit to the whole data set, including the data that will become part of the evaluation set (i.e., the OOB or testing set). Dropping *y* from the imputation models keeps the evaluation set blind to the outcome-covariate relationship in the training set. This is by default the case for *Val-MI*, where training and testing parts of the data set are imputed separately, so we did not consider *Val-MI(-y)*.

For comparison, we also analyzed data using simple *MI* and *MI(-y)* without internal validation. In addition, strategies were compared to internal validation (*Val*) in complete data, where possible. Since we did not observe changes in variability across the simulations when values were increased beyond *B*=10 and *M*=5,*B*=10 validation samples and *M*=5 imputations were used for BS and SS for incomplete data, and *B*=50 for complete data in the simulation studies. For CV, none (*B*=1) or *B*=5 repetitions and *M*=5 were used for incomplete, and *B*=1 or 25 repetitions for complete data. Note that these do not represent choices for *B* and *M* in practice, but that lower numbers can be used for simulation where variability across the 250 simulated data sets exceeds resampling and imputation variability within each data set.

### Modeling and performance measures

There is no unique definition for the performance of a prediction model. Three types of performance measures can be distinguished: measures of model *discrimination*, the ability of a model to separate outcome classes, i.e., to assign cases a higher risk than controls, measures of *calibration*, the unbiasedness of outcome predictions, in a way that of the observations with a predicted outcome probability of *pr*, about a fraction *pr* are cases, and measures of *overall performance*, the distance between observed and predicted outcome [3, 4].

We considered selected measures of each type for the binary (logistic) prediction model in the de novo simulation study. Of note, the focus was not on assessing the appropriateness of the different performance criteria in general, but rather to evaluate their estimation in the presence of missing values as compared to complete data.

As a discrimination measure, we considered the *area under the ROC curve* (AUC), which determines the probability that the model assigns a randomly chosen case (or, in more general terms, observation with outcome *y*=1) a higher predicted outcome probability than a randomly chosen control (observation with outcome *y*=0) and is equal to the concordance (*c*) statistic in the case of a binary outcome [4, 27]. As calibration measures, we used intercept and slope of a logistic regression model of observed against predicted outcomes, with deviation from 0 and 1, respectively, indicating suboptimal calibration [11, 28]. Finally, as overall performance measures we considered the *Brier score*, i.e., the average squared difference between observed and predicted outcomes, $\text {Brier} = \frac {1}{n}\sum _{i=1}^{n}{\left (y_{i} - \hat y_{i}\right)^{2}}$ [4, 29].

To assess added predictive performance of an extended as compared to a baseline model, we considered change in discrimination (*Δ*AUC) and three measures based on risk categories. These included, first, the *net reclassification improvement* (NRI), i.e., the difference between the proportion of observations moving into a ‘more correct’ risk category (i.e., cases moving up, controls moving down) and the proportion of observations moving into a ‘less correct’ risk category with the extended as compared to the baseline model [30]. This requires the definition of risk categories, where a single cutoff below the disease risk in the study population renders NRI by trend a measure for improvement in the classification of controls, and a single cutoff above the disease risk makes it a measure for improvement in the classification of cases [31]. In order to capture both, we chose three categories, [0, $\frac {1}{2}{frac}$], [$\frac {1}{2}{frac}, \frac {3}{2}{frac}$], [$\frac {3}{2}{frac}$,1], where *frac* (≤0.5, without loss of generality, since the NRI is not sensitive towards class label assignment) denotes the outcome case frequency in the data set (see Table 1 for simulation study). Second, we used the *continuous NRI*, a category-free version of the NRI [32], and lastly, the *integrated discrimination improvement* (IDI), which equals the integrated NRI over all possible risk cutoffs [30].

In the data-driven simulation study, the ability of inflammation-related markers to predict all-cause mortality was assessed using a Cox proportional hazards model, with and without additional inclusion of covariates known to be relevant for mortality prediction (age, sex, survey, BMI, systolic blood pressure, total to high density lipoprotein (HDL) cholesterol ratio, smoking status, alcohol intake and physical activity). To acknowledge potential non-linear effects, quadratic terms were additionally included for all continuous variables. We focused on one measure of discriminative model performance, namely time-dependent AUC at 10 years of follow-up according to the Kaplan-Meier method by Heagerty et al. [33]. Accordingly *Δ*AUC(10 years) was used as a measure of added predictive performance of the inflammation-related markers beyond the known predictors.

### Evaluation of competing strategies

In the *de novo* simulation study, the performance of the competing strategies of combining internal validation with imputation was assessed in terms of absolute bias, variance and mean squared error (MSE) of estimated performance criteria as compared to ‘true’ performance, defined as the average performance obtained when the model was fitted on the full (complete) data sets and evaluated on large (*n*=10,000) independent data sets with same underlying simulated effect sizes. Note that we did not compare (*Δ*)AUC estimates against the theoretical (*Δ*)AUC from which effect sizes were derived for simulation (see above), since these are often not achieved with small samples. In the data-driven simulation study, true effects were unknown. There, results of the competing strategies were compared against those from complete data.

### Construction of confidence intervals for performance estimates

Jiang et al. [34] proposed a simple concept to estimate confidence intervals for prediction errors in complete data. It is based on the numerical finding that the cross-validated prediction error asymptotically has the same variability as the apparent error. Thus, they suggest to construct confidence intervals for the prediction error by generating a percentile interval based on resampling for the apparent error and centering this interval at the prediction error. The underlying theory extends to other performance/precision measures [35]. Using the notation of the present manuscript, their proposed procedure follows the steps:

- Estimate the prediction error (point estimate) based on cross-validation (i.e. $\hat \theta ^{{Train,Test}}$).
- Conduct resampling (they suggest perturbation resampling, where random weights are assigned to the observations in each resampling step; for details we refer to their manuscript): For *b*=1,…,*B*, determine the resampling apparent error resulting from the resampled data (i.e. $\hat \theta ^{{BS(b),BS(b)}}$). Substract the original apparent error from the resampled one: $w_{b} = \hat \theta ^{{BS(b),BS(b)}} - \hat \theta ^{{Orig,Orig}}$.
- Obtain the *α*/2 and 1−*α*/2 percentiles $\hat \xi _{\alpha /2}$ and $\hat \xi _{1-\alpha /2}$ from the resampling distribution of the *w*_*b*_, *b*=1,…,*B*.
- Define the confidence interval for the prediction error as $\left [\hat \theta ^{{Train,Test}} - \hat \xi _{1-\alpha /2}\text {, } \hat \theta ^{{Train,Test}} + \hat \xi _{\alpha /2}\right ]$.

We modified the methodology with regard to several aspects. In step (2), we first used standard non-parametric bootstrapping as described above, and second, allowed for incomplete data by means of one of the combination strategies described above and in Fig. 2. That is, we obtained estimates $\hat \theta ^{{BS(b,m),BS(b,m)}}, b=1,\ldots,B, m=1,\ldots,M$, by fitting *and* evaluating the model in each (imputed) BS sample (i.e., in each BS sample that was imputed when strategy *Val-MI* was applied, or in each BS sample drawn from imputed data when strategy *MI-Val* was applied). For each *b* and *m*, we defined $w_{b,m} = \hat \theta ^{{BS(b,m),BS(b,m)}} - \hat \theta ^{{Orig,Orig}}$. In step (3), we obtained the *α*/2 and 1−*α*/2 percentiles from the empirical distribution of the *w*_*b*,*m*_, i.e. across all *B*×*M* estimates obtained. In step (4), we centered this interval at the BS 0.632+ estimate ($\hat \theta ^{0.632+}$) rather than the CV estimate $\hat \theta ^{{Train,Test}}$: $\left [\hat \theta ^{0.632+} - \xi _{1-\alpha /2}\text {,}\, \hat \theta ^{0.632+} - \xi _{\alpha /2} \right ]$, with *α*=0.05. The modified methodology can be integrated with performance estimation using the strategies described above within the same resampling (BS) scheme. For *Val-MI*, we performed *B*=100 bootstrap draws followed by *M*=1 imputation; for *MI(-y)-Val*, *M*=100 imputations were conducted followed by *B*=1 bootstrap draw. For complete data, *B*=100 was chosen. For comparison, we also constructed confidence intervals for apparent performance based on analytical test concepts, i.e., using DeLong’s test for AUC and *Δ*AUC. In the presence of missing values (strategies *MI* and *MI(-y)*), Rubin’s rules were applied to the AUC estimates and variances obtained from DeLong’s test [8].

### Software

All calculations were performed using R, version 3.0.1 [36]. Data generation involved use of the R package *mvtnorm*, version 0.9-9995 [37]. MICE was performed using the package *mice*, version 2.17 [6]. Internal validation was performed using custom code. For predictive performance measures, the R packages *pROC*, version 1.7.3 [38], *PredictABEL*, version 1.2-2 [39], and *survivalROC*, version 1.0.3 [40], were used. Example R code is available in Additional file 2.

## Results

### Importance of validation and comparative performance of validation strategies

**Fig. 3:**
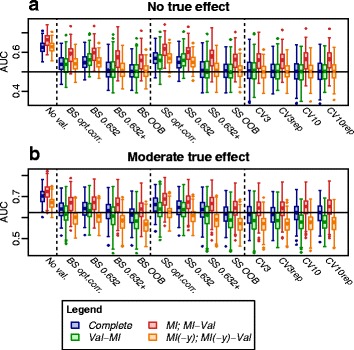
Simulation distribution of AUC estimates obtained by different strategies. *Boxplots* showing distribution of AUC estimates across the 250 simulated data sets in a setting with moderate sample size (*n*=200), *p*=10 covariates, moderate missing at random (MAR) missingness (*miss*=25 *%* of values missing), balanced outcome class distribution (*frac*=0.5) and uncorrelated covariates (*ρ*=0) in the absence (theoretical *auc*=0.5; **a**) and presence (theoretical *auc*=0.66, see text; **b**) of a moderate true effect of the covariates on the outcome. The *horizontal line* denotes ‘true’ AUC related to a complete data set of size 200 (which is not necessarily equal to theoretical *auc*; see text). *BS*, bootstrap; *CVK*, *K*-fold CV; *CVKrep*, repeated *K*-fold CV; *MI*, multiple imputation; *MI(-y)*, multiple imputation without including the outcome; *No val.*, no validation (i.e., apparent performance); *OOB*, out-of-bag estimate; *opt.corr.*, ordinary optimism-corrected estimate; *SS*, subsampling; *Val*, validation

In the *de novo* simulation experiment, complete and incomplete data were generated with varying data set characteristics, followed by applying the competing combined validation/imputation strategies. For comparison, we also assessed apparent performance, i.e., the performance in the original data in the case of complete data, and the performance estimates pooled using Rubin’s rules from MI in the case of incomplete data. Results are shown in Fig. 3 (for AUC, at *n*=200, *p*=10 covariates) and in Additional file 1: Figures S3 to S8 (for other performance measures and choices of parameters).

The apparent performance estimates were generally optimistic – even in the case of large sample size and small number of variables (Additional file 1: Figure S3; *n*=2000, *p*=1). Optimism was particularly strongly pronounced for imputed data when the outcome had been included in the imputation models (strategy *MI*).

Among the investigated ways to correct for optimism, the ordinary optimism correction and the 0.632 estimate tended to achieve less effective optimism control as compared to the BS/SS 0.632+ estimate, the BS/SS OOB estimate and CV estimates. This was most strongly observed in the absence of a true effect and with increasing number of covariates (Fig. 3 and in Additional file 1: Figures S4 to S8).

### Comparison of strategies of combining internal validation and multiple imputation

**Fig. 4:**
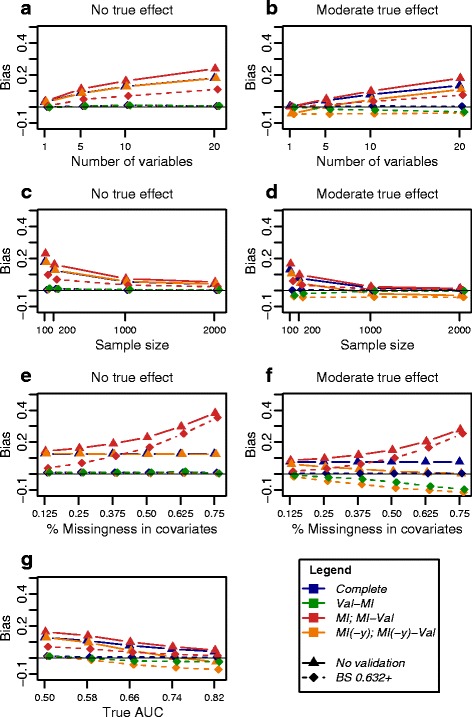
Bias of AUC estimates obtained by different strategies based on bootstrapping. Bias is shown for one varying data set characteristic in each panel (**a**, **b** number of covariates *p*; **c**, **d** sample size *n*; **e**, **f** degree of missingness *miss*; **g** true effect *auc*), while keeping all remaining characteristics constant: sample size (*n*=200), *p*=10 covariates, 25 % missing values, missing at random (MAR), balanced outcome class distribution (*frac*=0.5), uncorrelated covariates (*ρ*=0). Results are shown for absence (theoretical *auc*=0.5; **a**, **c**, **e**, **g**) and presence (theoretical *auc*=0.66; **b**, **d**, **f**, **g**) of a moderate true effect of the covariates on the outcome

The *MI-Val* strategy, i.e., conducting MI followed by internal validation (i.e., *BS*, *SS*, *CVK* or *CVKrep*) on the imputed data sets, generally yielded optimistically biased performance estimates and large mean squared errors in almost all settings, and more severely with an increasing number of variables, decreasing sample size, increasing degree of missingness, and decreasing true effect (shown for the AUC in Fig. 4 and in Additional file 1: Figure S9).

*MI(-y)-Val* was largely unbiased in the absence of a true effect, but gave pessimistic results when the covariates truely affected the outcome (Fig. 4), largely independent of the number of covariates and the sample size. A likely explanation is that omitting the outcome from the imputation disrupts the correlation structure among covariates and outcome, leading to underestimation of effect sizes. The pessimistic bias became more pronounced with increasing degree of missingness and increasing effect size.

*Val-MI* produced mostly unbiased AUC estimates; however, in the presence of a large number of missing values, a pessimistic bias was observed in the presence of a true underlying effect (Fig. 4). This trend was mostly weaker than for the *MI(-y)-Val* strategy and depended also on sample size, number of covariates and true effect size.

Varying other data set characteristics, such as missingness mechanism, outcome class frequencies, correlation among the variables, number of baseline covariates and degree of missingness among baseline covariates, did not greatly influence results (Additional file 1: Figures S10 and S15).

### Trends observed for different model performance measures

Although focusing on the AUC as a discrimination measure, the above described trends were largely similar across the model performance measures investigated (Additional file 1: Figures S11 to S21). Of note, biases that were already present in complete data were found to be mirrored, and sometimes augmented, in incomplete data. Examples include the negative bias of *Δ*AUC (Additional file 1: Figures S13 and S15) and the positive bias of categorical NRI (Additional file 1: Figure S16) in the absence of a true effect, specifically with increasing number of covariates and decreasing sample size. Another example is the pessimistic bias of the Brier score that was most strongly observed for *Val-MI* with increasing degree of missingness, number of covariates and decreasing sample size. Importantly, both *Val-MI* and *MI(-y)-Val* strategies generally did not produce (optimistic) bias that was not already (at least to a weaker extent) observed in complete data results.

In terms of calibration, models tended to be miscalibrated in test (OOB) data for most strategies in both complete and incomplete data (Additional file 1: Figures S22 to S27). This trend became worse with decreasing number of covariates and was often observed such that calibration lines were too steep (i.e., intercept <0; slope >1), rendering recalibration of prediction models a desirable step. Although not influencing discriminative test performance, this might improve overall test performance (as measured e.g. by the Brier score).

### Extension to a real-data situation

In order to assess how the competing strategies of combining internal validation and MI performed in a realistic situation, we based another simulation experiment on a real data set. In the population-based MONICA/KORA subcohort, the aim was to assess the ability of blood concentrations of inflammatory markers for predicting all-cause mortality over a follow-up time of 15 years in *n*=2012 healthy adults. We used the 1258 complete observations as a basis for a data-driven simulation study, where we imposed missingness on these data in a way that reflected the missingness structure in the original incomplete data set (Additional file 1: Figure S1), followed by applying the competing combined validation/imputation strategies to obtain time-dependent (change) in AUC.

**Fig. 5:**
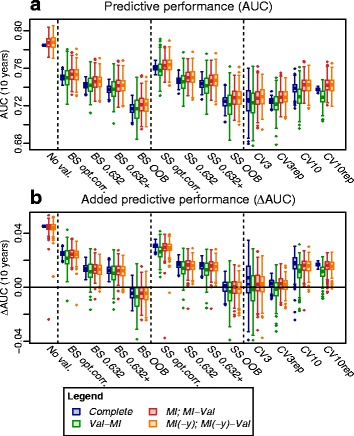
Data-driven simulation. *Boxplots* showing distribution of performance estimates across the 250 simulations of missing values into the complete-observations data set derived from the MONICA/KORA subcohort. **a**, AUC (10 years), performance of a model comprising the 15 inflammation-related markers; **b**, *Δ*AUC (10 years), added performance of the inflammation-related markers on top of the baseline covariates. Note that the variability across the 250 simulations reflects variability in imposing missing values as well as resampling variability, but not population variability as is part of the variability in Fig. 3. *BS*, bootstrap; *CVK*, *K*-fold CV; *CVKrep*, repeated *K*-fold CV *MI*, multiple imputation; *MI(-y)*, multiple imputation without including the outcome; *No val.*, no validation (i.e., apparent performance); *OBB*, out-of-bag estimate; *opt.corr.*, ordinary -corrected estimate; *SS*, subsampling; *Val*, validation

Results are shown in Fig. 5. Without validation, performance estimates were much higher than those obtained with validation, confirming the importance of validation for assessment of predictive performance. With ordinary optimism correction, performance estimates were still higher than for the other estimates, in line with the assumption that it may achieve insufficient correction for optimism. The lowest values were observed for the OOB, CV3 and CV3rep estimates, suggesting a pessimistic bias, which seemed to be improved by the 0.632+ estimates.

Differences between the strategies of combining validation and imputation were less pronounced, presumably due to the large sample size and small proportion of missing values (7.2 % on average among the inflammation-related markers). *Val-MI* yielded lower *Δ*AUC estimates on average as compared to *Val* on complete data. This was consistent with our observation of a slight pessimism of *Val-MI* in the de novo simulation study in the presence of a true effect, and was even more strongly observed for *CV10* and *CV10rep*. *Val-MI* also appeared more variable as compared to the other strategies. This is likely due to the fact that at the given low proportion of missing values, e.g. performing *B*=10 BS first followed by *M*=5 imputations on each yields less distinct data sets than performing *M*=5 imputations first followed by *B*=10 random BS runs or performing *B*=50 BS runs on the complete data.

### Choice of number of resamples and number of imputations in practice

We addressed the question of how large the number of resamples *B* and the number of imputations *M* should be chosen in practice, for two of the best-performing strategies, *Val-MI* and *MI(-y)-Val* based on bootstrapping with the 0.632+ estimate. Therefore, we repeated the de novo simulation study for selected parameter settings with varying *B* and *M*.

**Fig. 6:**
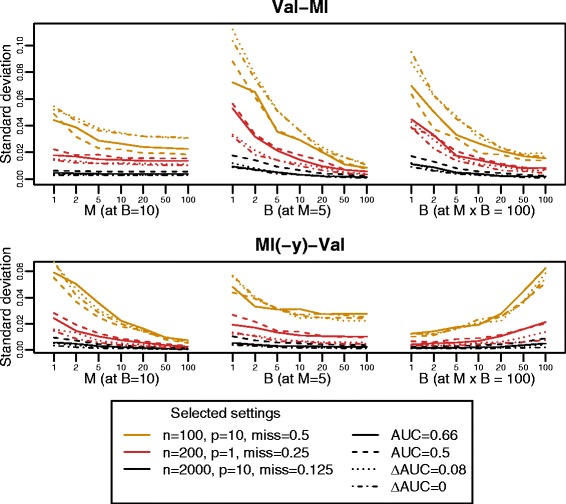
Choice of number of resamples *B* and imputations *M* in practice. Standard deviation of performance estimate (AUC for performance, *Δ*AUC for added performance) across 10 runs, averaged across 10 simulated data sets for strategies *Val-MI* and *MI(-y)-Val* based on bootstrapping at varying *B* and *M*. Apart from the parameters provided in the legend, parameters were chosen as in Fig. 3. In the case of added performance, the number of variables in the baseline model was set to *p*_0_=1

For *Val-MI*, we observed a steep decline of variability of performance estimates with increasing *B*, where as decline was weaker with increasing *M* (Fig. 6). This is expected, especially in the settings with lower degree of missingness, where the imputed data sets are not expected to differ strongly from each other. At constant total number *B*·*M*, the best option seems to be to choose the largest possible value of *B* (with *M*=1). This is also not unexpected, given that imputation variability is added on top of resampling variability in each sample.

In contrast, for *MI(-y)-Val*, *M* seemed to be the number that mostly determined variability, with variability decreasing with increasing *M* even at constant *B*·*M* (Fig. 6). Furthermore, variability of performance estimates was generally larger in *Val-MI* as compared to *MI(-y)-Val*, even with the least variable combination of *B* and *M* at constant total number *B*·*M*.

Thus, it is recommendable to choose *B* and *M* as large as possible if applying *Val-MI* and *MI(-y)-Val*, respectively. An analytic relationship can be utilized in order to assess variability of performance estimates with increasing *B* and *M*, respectively: The standard deviation of the mean is generally equal to the population standard deviation divided by the square root of the sample size, given that values are independent. Since the *B* performance estimates obtained with e.g. *Val-MI* are independent with regard to the BS, we can assume that the following relationship holds:

2 $$ \text{SD}\left(\hat\theta_{B} \right) = \frac{1}{\sqrt{B}} \text{SD}\left(\hat\theta_{1}\right),  $$

whereby SD denotes the standard deviation, and $\hat \theta _{B}$ the performance estimate when *B* resamples were conducted (and *M*=1 imputations). Empirical evidence confirms this assumption for both *Val-MI* and *MI(-y)-Val* (Additional file 1: Figures S28 and S29). Thus, we provide standard deviation estimates at *B*=1 and *M*=1 for various parameter settings in Additional file 1: Tables S2 and S23. This may allow the reader to approximate the standard deviation for their situations at larger values of *B* or *M* using Eq. (2) and to choose *B* or *M* such that the required accuracy is obtained.

### Incomplete future patient data

In the context of building prediction models in the presence of missing values, it has been noted earlier that future patients, to which the prediction model will be applied, might not have complete data for all covariates in the model [13]. To still allow application of the model, the missing values might be imputed using a set of patient data, whereby, notably, the outcome variable is not available. Thus, a relevant question that arises is whether and how predictive performance suffers from missingness in the evaluation data. Therefore, we evaluated models fitted to simulated complete data in large independent data sets with the same underlying simulated effect sizes and varying degrees of missingness, imputed using *MI(-y)*. We observed a clear decrease of predictive performance when the proportion of missing values in the test data increased (Additional file 1: Figure S30). This was observed most severely (in absolute terms) with larger true performance.

### An approach towards confidence intervals for performance estimates

**Fig. 7:**
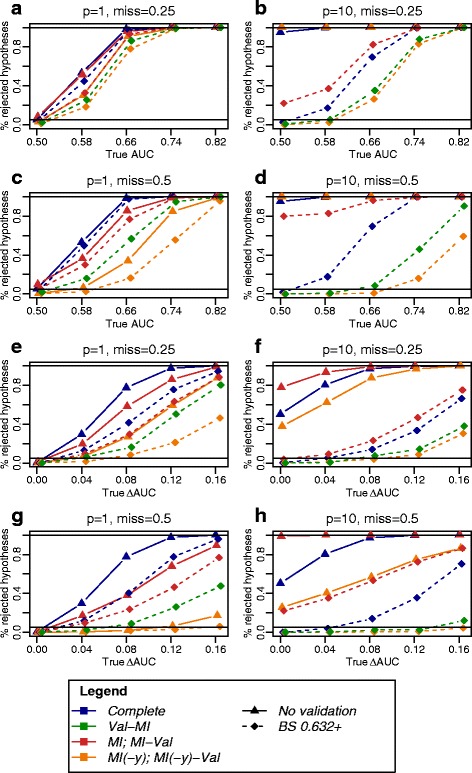
Type 1 error and power of resampling-based confidence intervals for AUC and *Δ*AUC estimates. Percentage of rejected null hypotheses (i.e., confidence interval above 0.5 and 0 for AUC (**a**, **b**, **c**, **d**) and *Δ*AUC, (**e**, **f**, **g**, **h**) respectively) among 250 simulations plotted against the underlying true (theoretical) value. In the absence of a true effect (true *auc*=0.5; *Δ* *auc*=0), percentage of rejected null hypotheses equals type 1 error, otherwise power. Parameters were chosen as denoted in the figure titles, *n*=200,*p*_0_=0,1 and otherwise as in Fig. 3

As an outlook, we considered an approach of constructing resampling-based confidence intervals for performance estimates that is based on the work by Jiang et al. [34]. Figure 7 shows type 1 error and power for AUC and *Δ*AUC estimates for the competing strategies. Thereby, type 1 error was defined as the proportion of simulations with true AUC =0.5 or *Δ*AUC=0, where a test with the null hypothesis AUC =0.5 or *Δ*AUC=0 was rejected (i.e., confidence interval above 0.5 and 0, respectively). In the presence of a true effect (AUC > 0.5 or *Δ*AUC>0), this proportion specified power. In a low-dimensional situation (*p*=1), the nominal type 1 error rate of 5 % was kept on average for all strategies (Fig. 7 a, c, e, g). However, at *p*=10 severely inflated type 1 error rates were observed for the strategies without validation (i.e., based on DeLong’s test) and for the *MI-Val* 0.632+ estimate, while in complete data, *Val-MI* and *MI(-y)-Val*, the 0.632+ estimate kept the nominal type 1 error rate (Fig. 7 b, d, f, h). As expected, the presence of missing values diminished power, as observed for *Val-MI* as compared to *Val* on complete data, and to an even stronger extent for *MI(-y)-Val*. Together, the proposed approach proposes to be a way of obtaining valid confidence intervals for both *Val-MI* and *MI(-y)-Val* 0.632+ estimates without additional computational costs.

## Discussion

Using simulated and real data we have compared strategies of combining internal validation with multiple imputation in order to obtain unbiased estimates of various (added) predictive performance measures. Our investigation covered a wide range of data set characteristics, validation strategies and performance measures, and also dealt with practical questions such as the numbers of imputations and bootstrap samples to be chosen in a given data set, and the aspects of incomplete future patient data and the construction of confidence intervals for performance estimates.

Throughout the investigated simulation settings, we observed an optimistic bias for apparent performance estimates, which was insufficiently corrected by ordinary optimism correction and the BS (and SS) 0.632 estimate, whereas the OOB estimate tended to be pessimistic and the 0.632+ tended to provide unbiased estimates. CV estimates were more variable than BS estimates (although this comparison might not be completely fair since the total number of training/test set pairs was not always the same in BS/SS as in CV or CVrep). These trends were similarly observed for complete and incomplete data and are consistent with previous observations for complete data. For instance, Wehberg and Schumacher [41] reported the 0.632+ method to outperform ordinary optimism correction and 0.632, while the OOB estimate was pessimistic. Also, Smith et al. [1] and Braga-Neto et al. [42] observed insufficient optimism correction for the ordinary method and the 0.632 estimate, respectively, and both reported increased variability of CV estimates. Another publication focused on AUC estimation and found the BS 0.632+ estimate to be the least biased and variable one among the BS estimates [43].

When we investigated strategies of combining validation with imputation, we observed an optimistic bias for the strategy of imputing first and then resampling on the imputed data (*MI-Val*), whereas imputing training and test sets separately (*Val-MI*) provided largely unbiased and sometimes pessimistic results. The question of in which order bootstrapping and imputation should be combined has been studied before from a theoretical [44] and empirical [12] perspective. In *MI-Val*, all observations, which are later on repeatedly separated into training (BS) and test (OOB) sets, are imputed in one imputation process. Since values are imputed using predictions based on multivariate models including all observations, it is evident that future test observations do not remain completely blind to future training observations. Still, the severity of the expected optimism of the *MI-Val* approach given different data characteristics, validation strategies and performance estimates has not been intensively studied. In practice, both *MI-Val* and *Val-MI* have been applied before [9, 10, 45].

*Val-MI* tended to be pessimistically biased in the presence of a true underlying effect in our and others’ [12] work. Specifically, when sample size is low and number of covariates large, the model overfits the training (BS) part of the data set, resulting in a worse fit to the test (OOB) data. In the presence of missing values, training and test data are imputed separately. It can be assumed that overfitting also occurs at the stage of imputation (where imputation models might become overfitted to the observed data both in the training and in the test set). This may result in a more severe difference in the observed covariate-outcome relationships between training and test data, and consequently worse fit of the model fitted to the training data to the test data, yielding an underestimation of predictive performance that apparently cannot be fully corrected using the 0.632+ estimate.

*MI(-y)-Val* produced mostly pessimistic results in the presence of an underlying true effect, mostly independent of sample size and number of covariates. In general MI literature, it is not recommended to omit the outcome from the imputation models [26, 46]. Omitting the outcome equals making the assumption that it is not related with the covariates, as stated by von Hippel [26]. This assumption is wrong in the case of a true underlying effect, resulting in misspecified imputation models, and, in turn, in an underestimation of effect estimates [46]. Of note, the same study reported no difference between the *MI* and *MI(-y)* methods as far as inference is concerned. To our knowledge, the issue has not been investigated in the context of predictive performance estimation. In their study of ‘incomplete’ CV, Hornung et al. [14] investigated the effect of – amongst other preprocessing steps – imputing the whole data set prior to CV as compared to basing the imputation on the training data only. They used a single imputation method that omitted the outcome, and found only little impact on CV error estimation.

For measures of added predictive performance we made the observation that even in complete data, estimates were sometimes biased in the absence of a true effect. For instance, *Δ*AUC and categorical NRI were pessimistically and optimistically biased, respectively. The optimistic bias of NRI has lead to critical discussion [47]. It is not unexpected that such bias is not eliminated when the respective validation method is combined with imputation.

Our study focused on treating missing values and deriving reasonable estimates for predictive performance measures in the presence of incomplete data in the model development phase, i.e., in the phase where complete outcome data are available and one aims to derive a prediction model for use in future data.

Our study focused on treating missing values and deriving reasonable estimates for predictive performance measures in the presence of incomplete data in the *research stage*, i.e. in the situation where data sets with complete outcome data are available from studies/cohorts and one aims to develop a prediction model for use in future patient data (as opposed to the *application stage* where the model is applied to predict patients’ outcome). Thus, when we evaluated estimates, they were compared against average performance in large complete data sets. An important question is how missing values in future patient data impair the performance of a developed prediction model, and whether such impairment would have to be considered already when developing the model. It has been suggested that data in the research stage should be imputed omitting the outcome from the imputation process, at least in the test sets, to get close to the situation in future real-world clinical data, where no outcome would be available for imputation either [13]. According to this suggestion, the strategy *Val-MI* should be avoided. However, how close a predictive performance estimate obtained through any strategy on the research data approximates the actual performance in future clinical data, depends strongly on the similarity in the proportion (and putatively, in the pattern) of missing values in both situations. Our and others’ [48] results suggest that – irregardless of how missing values in future clinical data are treated – accuracy is lost with increasing missingness in future data at a given proportion of missingness in the research data. We expect the proportion of missing values in future patient data to be lower than that in study data in many cases. Specifically, epidemiological study data are subject to additional missingness attributable to design, sample availability and questionnaire response. Since the precise missingness patterns in both study data and future patient data in clinical practice may vary between studies and the outcome of interest, no general rule can be developed for estimating predictive performance of a model when future patient data are expected to contain missing values.

We propose a simple integrated approach for the construction of confidence intervals for performance estimates. The resulting intervals kept the nominal type 1 error rate for both *Val-MI* and *MI(-y)-Val*, although a severe loss in power as compared to complete data could be observed. The chosen approach relies on the numerical finding that prediction error estimates have the same variability as apparent error estimates and thus, bootstrap intervals for apparent error can be centered at prediction error estimate [34]. The strategy has a major computational advantage over alternative strategies of constructing confidence intervals for estimates of prediction error/performance measures that use resampling in order to estimate the distribution of e.g. CV errors [49]. The latter require nesting the whole validation (and imputation) procedure within an outer resampling loop. Other alternatives that do not require a double resampling loop might rely on tests applied to the test data. An example is the median *P* rule suggested by van de Wiel et al. [50], where a nonparametric test is conducted on the test parts of a subsampling scheme, resulting in a collection of *P* values of which the median is a valid summary that controls the type 1 error under fairly general conditions. The methodology could be generalized to other (parametric or nonparametric) tests conducted on the test observations, such as DeLong’s test for (*Δ*)AUC, and extension to incomplete data is possible with the help of Rubin’s combination rules. However, this strategy might lack power, because tests are conducted on the small test sets.

Together, our findings allow the careful formulation of recommendations for practice. First, if one aims to assess predictive performance of a model, validation is of utmost importance to avoid overoptimism. As for complete data, bootstrap with the 0.632+ estimate, turned out to be a preferable validation strategy also in the case of incomplete data. When combining internal validation and MI, one should not impute the full data set including the outcome in the imputation followed by resampling (strategy *MI-Val*) due to its optimistic bias. Instead, we can recommend nesting the MI in the resampling (*Val-MI*) or performing MI first, but without including the outcome variable (*MI(-y)-Val*). The number of resamples (*B*) and imputations (*M*) should be maximized in *Val-MI* and *MI(-y)-Val*, respectively. The choice of exact number of resamples and imputations for a given data set can be guided by the variability data we provide. In many situations and for many performance criteria, *Val-MI* might be preferable, although this choice may also depend on computational capacity, which is lower for *MI(-y)-Val*, where variability of the 0.632+ estimate is lower at the same number of resamples and only half the number of imputation runs is required. One should also be aware of (complete-data) biases of specific performance criteria, which may be augmented in the presence of missing values. Finally, one possible way of constructing valid confidence intervals for predictive performance estimates may be to center the bootstrap interval of the apparent performance estimate at the predictive performance estimate. This strategy can be easily embedded in the *Val-MI* and *MI(-y)-Val* strategies.

Strengths of this study include its comprehensiveness with regard to different data characteristics, validation strategies and performance measures, and the use of both simulated and real data. Our investigation may be extended with regard to several aspects. For instance, we did not vary effect strengths between the covariates. The relationship between effect strengths and missingness in covariates may influence the extent of potential bias in e.g. *Val-MI*. Furthermore, it will be interesting to extend the study on confidence intervals by adopting alternative approaches to incomplete data, with a focus on searching for a strategy that improves power. In addition, one might explore the role of the obtained findings in a higher-dimensional situation where variable selection and parameter tuning often requires an inner validation loop. Of note, while in our study results were very similar for BS and SS, in an extended situation involving model selection, or hypothesis tests following [50], SS should be preferred due to known flaws of the BS methodology [51].

## Conclusions

In the presence of missing values, our most recommendable strategy to obtain estimates of predictive performance measures is to perform bootstrap for internal validation, with separate imputation of training and test parts and to determine the 0.632+ estimate. For this strategy, at given computational capacity, the number of resamples should be maximized. The strategy allows for the integrated calculation of confidence intervals for the performance estimate.

## Additional files

Additional file 1 Supplementary Figures and Tables. **Figure S1.** Imposing missingness into complete observations from the application data set. **Figure S2.** Visualization of internal validation strategies in complete data. **Figure S3.** Simulation distribution of AUC estimates obtained by different strategies at large sample size (*n*=2000) and *p*=1 covariate. **Figure S4.** Simulation distribution of AUC estimates obtained by different strategies at large sample size (*n*=2000) and *p*=1 covariate. **Figure S5.** Simulation distribution of *Δ*AUC estimates obtained by different strategies. **Figure S6.** Simulation distribution of categorical NRI estimates obtained by different strategies. **Figure S7.** Simulation distribution of continuous NRI estimates obtained by different strategies. **Figure S8.** Simulation distribution of IDI estimates obtained by different strategies. **Figure S9.** Mean squared error of AUC estimates obtained by different strategies based on bootstrapping. **Figure S10.** Bias of AUC estimates obtained by different strategies based on bootstrapping – Influence of further data characteristics. **Figure S11.** Bias of Brier score estimates obtained by different strategies based on bootstrapping. **Figure S12.** Mean squared error of Brier score estimates obtained by different strategies based on bootstrapping. **Figure S13.** Bias of *Δ*AUC estimates obtained by different strategies based on bootstrapping. **Figure S14.** Mean squared error of *Δ*AUC estimates obtained by different strategies based on bootstrapping. **Figure S15.** Bias of *Δ*AUC estimates obtained by different strategies based on bootstrapping – Influence of further data characteristics. **Figure S16.** Bias of categorical net reclassification improvement (NRI) estimates obtained by different strategies based on bootstrapping. **Figure S17.** Mean squared error of categorical net reclassification improvement (NRI) estimates obtained by different strategies based on bootstrapping. **Figure S18.** Bias of continuous net reclassification improvement (NRI) estimates obtained by different strategies based on bootstrapping. **Figure S19.** Mean squared error of continuous net reclassification improvement (NRI) estimates obtained by different strategies based on bootstrapping. **Figure S20.** Bias of continuous integrated discrimination improvement (IDI) estimates obtained by different strategies based on bootstrapping. **Figure S21.** Mean squared error of integrated discrimination improvement (IDI) estimates obtained by different strategies based on bootstrapping. **Figure S22.** Simulation distribution of calibration intercept estimates obtained by different strategies. **Figure S23.** Bias of calibration intercept estimates obtained by different strategies based on bootstrapping. **Figure S24.** Mean squared error of calibration intercept estimates obtained by different strategies based on bootstrapping. **Figure S25.** Simulation distribution of calibration slope estimates obtained by different strategies. **Figure S26.** Bias of calibration slope estimates obtained by different strategies based on bootstrapping. **Figure S27.** Mean squared error of calibration slope estimates obtained by different strategies based on bootstrapping. **Figure S28.** Relationship of standard deviations of performance estimates with varying number of resamples and imputations. **Figure S29.** Standard deviation of performance estimates at varying number of resamples and imputations. **Figure S30.** Impairment of performance evaluation through missing values in the test data. **Table S1.** Descriptive information of phenotypic and inflammation-related markers from the MONICA/KORA subcohort. **Table S2.** Standard deviation of performance estimates obtained with *Val-MI* at one resample (*B*=1) and one imputation (*M*=1). **Table S3.** Standard deviation of performance estimates obtained with *MI(-y)-Val* at one imputation (*M*=1) and one resample (*B*=1). (PDF 1823 kb)

Additional file 2 R code. Example_R_Code.r Example R code showing how to apply functions. gen.data.r R functions for generation of data and imposing missingness. do.resampling.r R functions for performing internal validation and multiple imputation. get.performance.r R functions to do the modeling and obtain performance estimates. do.CIs.r R functions to obtain point and confidence interval estimates. (ZIP 20 kb)

## Abbreviations

*Δ*AUC: Change in AUC
AUC: Area under the ROC curve
BS: Bootstrap
CV: Cross-validation
CVK: *K*-fold cross-validation
CVKrep: Repeated *K*-fold cross-validation
HDL: High density lipoprotein
IDI: Integrated discrimination improvement
KORA: Cooperative health research in the region of Augsburg
MAR: Missing at random
MARblock: Blockwise missing at random
MCAR: Missing completely at random
MI: Multiple imputation
MI-Val: Multiple imputation followed by internal validation
MI(-y): Multiple imputation without including the outcome in the imputation models
MI(-y)-Val: MI(-y) followed by internal validation
MICE: Multiple imputation by chained equations
MONICA: Monitoring trends and determinants in cardiovascular disease
NRI: Net reclassification improvement
OOB: Out-of-bag
ROC: Receiver-operating characteristic
SS: Subsampling
Val: Validation
Val-MI: Internal validation followed by multiple imputation
