# Erratum to: Assessment of predictive performance in incomplete data by combining internal validation and multiple imputation

**DOI:** 10.1186/s12874-016-0271-7

**Published:** 2016-12-05

**Authors:** Simone Wahl, Anne-Laure Boulesteix, Astrid Zierer, Barbara Thorand, Mark A. van de Wiel

**Affiliations:** 1 Research Unit of Molecular Epidemiology, Helmholtz Zentrum München - German Research Center for Environmental Health, Ingolstädter Landstrasse 1, 85764 Neuherberg, Germany; 2 Institute of Epidemiology II, Helmholtz Zentrum München - German Research Center for Environmental Health, Ingolstädter Landstrasse 1, 85764 Neuherberg, Germany; 3 German Center for Diabetes Research (DZD e.V.), Ingolstädter Landstrasse 1, 85764 Neuherberg, Germany; 4 Department of Medical Informatics, Biometry and Epidemiology, Ludwig-Maximilians-Universität München, Marchioninistrasse 15, 81377 Munich, Germany; 5 Department of Epidemiology and Biostatistics, VU University Medical Center, PO Box 7057, 1007 Amsterdam, MB The Netherlands; 6 Department of Mathematics, VU University, De Boelelaan 1081a, 1081 Amsterdam, HV The Netherlands

## Erratum

After publication of the original article [1], it came to the authors’ attention that Mark A. van de Wiel’s name was spelled incorrectly due to a misinterpreted correction submitted at proofing stage. The author’s middle initial and family name had been combined inadvertently.

Mark A. van de Wiel’s name is spelled correctly in this erratum, and the original article has been updated to reflect this correction.
