# Supplementary material for: Assessment of predictive performance in incomplete data by combining internal validation and multiple imputation
Source: BMC Med Res Methodol. 2016 Oct 26;16:144. doi: 10.1186/s12874-016-0239-7 (PMC5080703; doi:10.1186/s12874-016-0239-7)
Supplement: Additional file 1 — Supplementary Figures and Tables. Figure S1. Imposing missingness into complete observations from the application data set. Figure S2. Visualization of internal validation strategies in complete data. Figure S3. Simulation distribution of AUC estimates obtained by different strategies at large sample size (n=2000) and p=1 covariate. Figure S4. Simulation distribution of AUC estimates obtained by different strategies at large sample size (n=2000) and p=1 covariate. Figure S5. Simulation distribution of ΔAUC estimates obtained by different strategies. Figure S6. Simulation distribution of categorical NRI estimates obtained by different strategies. Figure S7. Simulation distribution of continuous NRI estimates obtained by different strategies. Figure S8. Simulation distribution of IDI estimates obtained by different strategies. Figure S9. Mean squared error of AUC estimates obtained by different strategies based on bootstrapping. Figure S10. Bias of AUC estimates obtained by different strategies based on bootstrapping – Influence of further data characteristics. Figure S11. Bias of Brier score estimates obtained by different strategies based on bootstrapping. Figure S12. Mean squared error of Brier score estimates obtained by different strategies based on bootstrapping. Figure S13. Bias of ΔAUC estimates obtained by different strategies based on bootstrapping. Figure S14. Mean squared error of ΔAUC estimates obtained by different strategies based on bootstrapping. Figure S15. Bias of ΔAUC estimates obtained by different strategies based on bootstrapping – Influence of further data characteristics. Figure S16. Bias of categorical net reclassification improvement (NRI) estimates obtained by different strategies based on bootstrapping. Figure S17. Mean squared error of categorical net reclassification improvement (NRI) estimates obtained by different strategies based on bootstrapping. Figure S18. Bias of continuous net reclassification improvement (NRI) estimates obtained [file 12874_2016_239_MOESM1_ESM.pdf]

Assessment of predictive performance in incomplete data by combining internal validation and multiply imputation

Simone Wahl, Anne-Laure Boulesteix, Astrid Zierer, Barbara Thorand, Mark van de Wiel

Additional file 1: Supplementary Figures and Tables

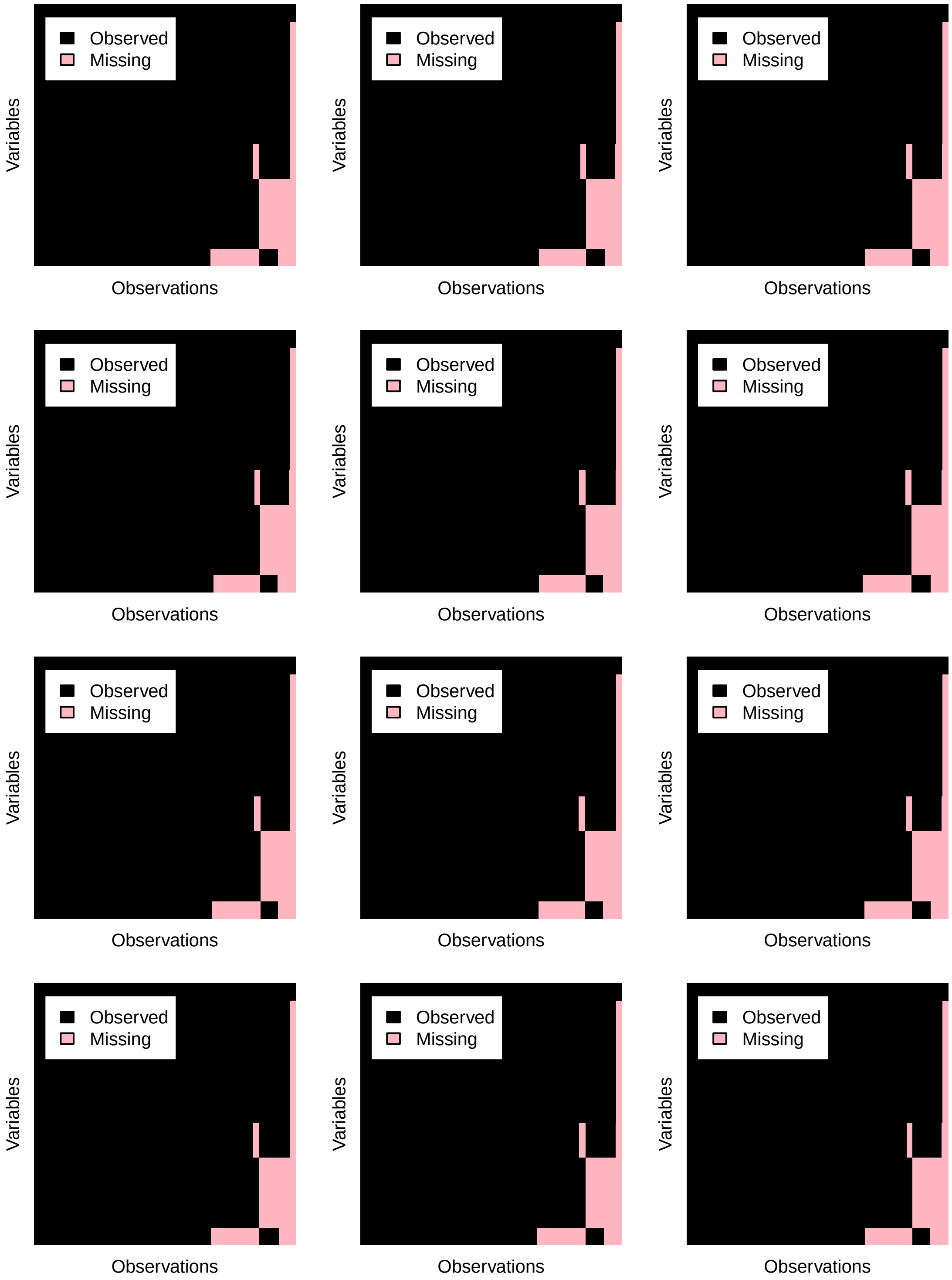

Figure S1: Imposing missingness into complete observations from the application data set. Missingness pattern among inflammation-related markers after missingness was imposed to complete observations in a way that the original missingness pattern is approximated. Shown for 12 randomly chosen simulations. Plot of missingness indicators (black=entry observed; red=entry missing) for the 1258 observations against the 15 inflammation-related markers, both sorted by missingness.

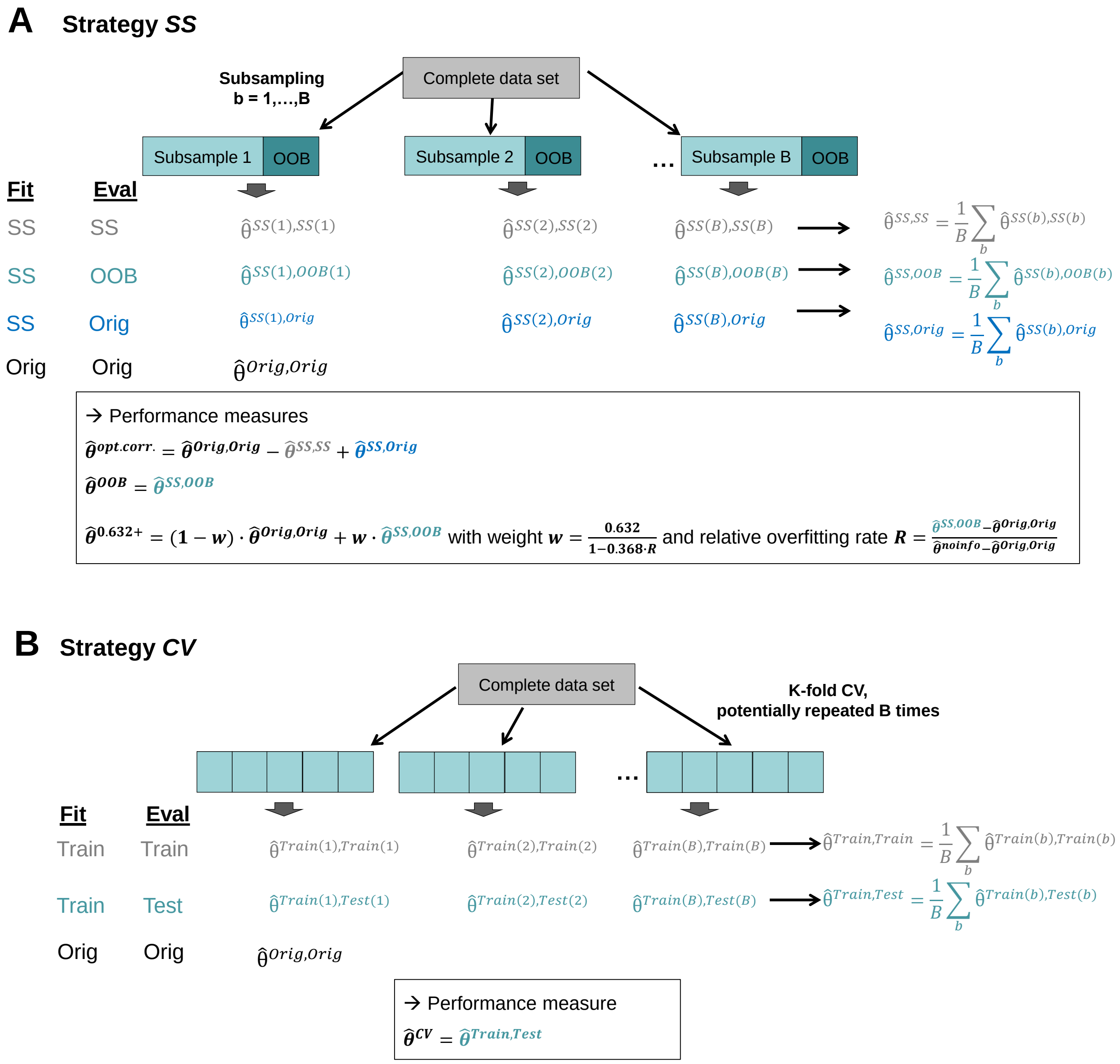

Figure S2: Visualization of internal validation strategies in complete data. **A** Subsampling (SS); **B** Cross-validation (CV). $\hat{\theta}^{Dat_1,Dat_2}$ denotes average performance across the $B \cdot M$ sets of data for model fitted on Dat_1 and evaluated on Dat_2, with $Orig$ denoting original (completed) data set, BS denoting BS sample and OOB denoting OOB sample. $\hat{\theta}^{noinfo}$: performance in the absence of an effect (see description in main text). **Performance measures**: $\hat{\theta}^{opt.corr.}$, ordinary optimism-corrected BS estimate; $\hat{\theta}^{OOB}$, OOB performance estimate; $\hat{\theta}^{0.632+}$, BS 0.632+ estimate. In the specific case of $w = 0.632$, the BS 0.632 estimate is obtained. $\hat{\theta}^{CV}$, CV performance estimate.

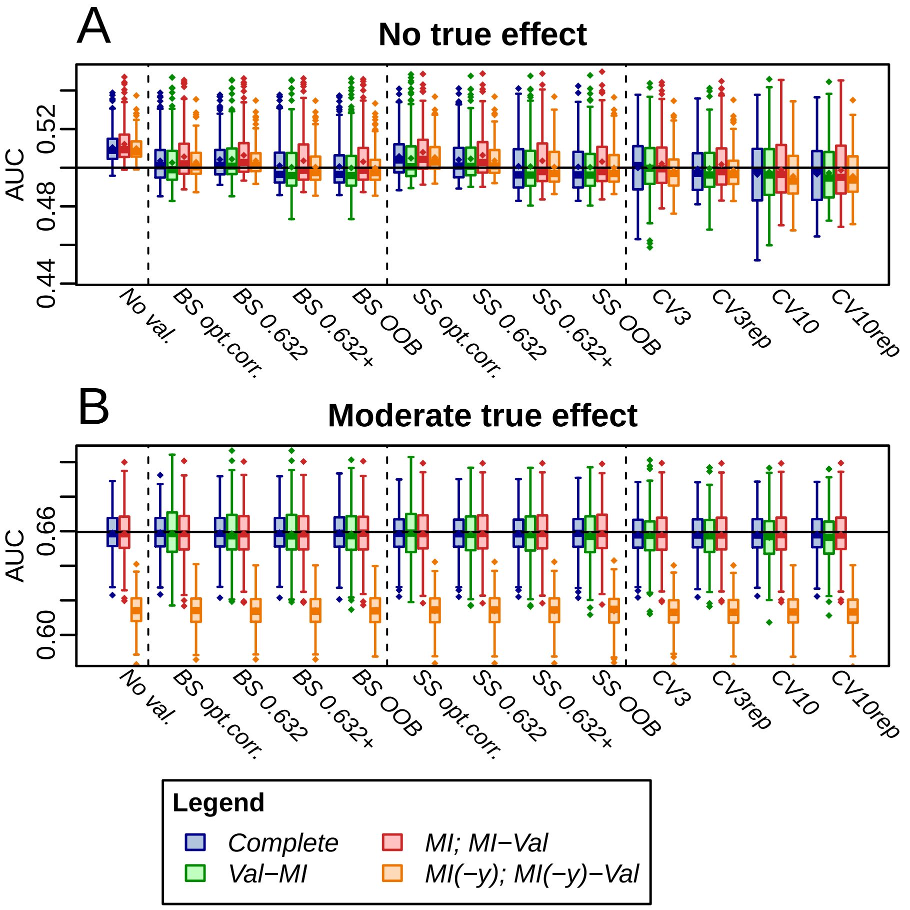

Figure S3: Simulation distribution of AUC estimates obtained by different strategies at large sample size ($n = 2000$) and $p = 1$ covariate. Boxplots showing distribution of Brier score estimates across the 250 simulated data sets in a setting with large sample size ($n = 2000$), $p = 1$ covariate, moderate missing at random (MAR) missingness ($miss = 25\%$ of values missing), balanced outcome class distribution ($frac = 0.5$) and uncorrelated covariates ($\rho = 0$) in the absence (theoretical $auc = 0.5$; **A**) and presence (theoretical $auc = 0.66$; **B**) of a moderate true effect of the covariates on the outcome. The horizontal line denotes 'true' AUC related to a complete data set of size 2000 (which is not necessarily equal to theoretical auc; see text). *BS*, bootstrap; *CVK(rep)*, (repeated) K-fold CV; *MI*, multiple imputation; *MI(-y)*, multiple imputation without including the outcome; *No val.*, no validation (i.e., apparent performance); *OOB*, out-of-bag estimate; *opt.corr*, ordinary optimism-corrected estimate; *SS*, subsampling; *Val*, validation.

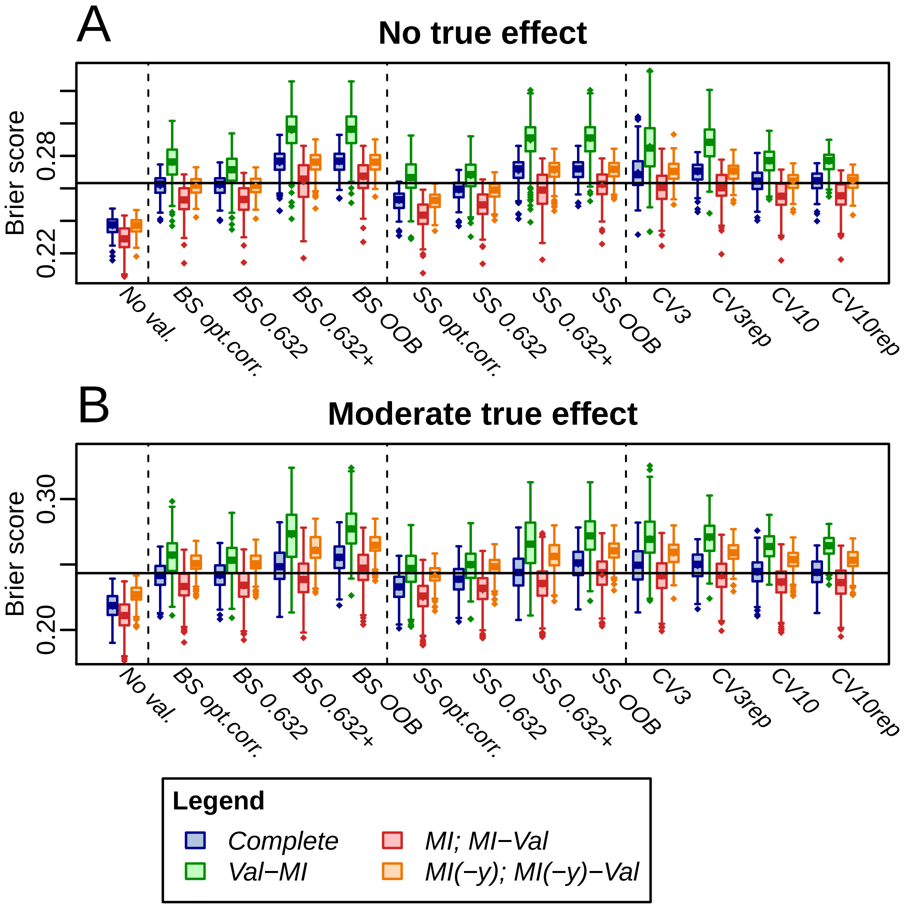

Figure S4: Simulation distribution of Brier score estimates obtained by different strategies. Boxplots showing distribution of Brier score estimates across the 250 simulated data sets in a setting with moderate sample size ($n = 200$), $p = 10$ covariates, moderate missing at random (MAR) missingness ($miss = 25\%$ of values missing), balanced outcome class distribution ($frac = 0.5$) and uncorrelated covariates ($\rho = 0$) in the absence (theoretical $auc = 0.5$; **A**) and presence (theoretical $auc = 0.66$; **B**) of a moderate true effect of the covariates on the outcome. The horizontal line denotes 'true' Brier score related to a complete data set of size 200 (see text). *BS*, bootstrap; *CVK(rep)*, (repeated) K-fold CV; *MI*, multiple imputation; *MI(-y)*, multiple imputation without including the outcome; *No val.*, no validation (i.e., apparent performance); *OOB*, out-of-bag estimate; *opt.corr*, ordinary optimism-corrected estimate; *SS*, subsampling; *Val*, validation.

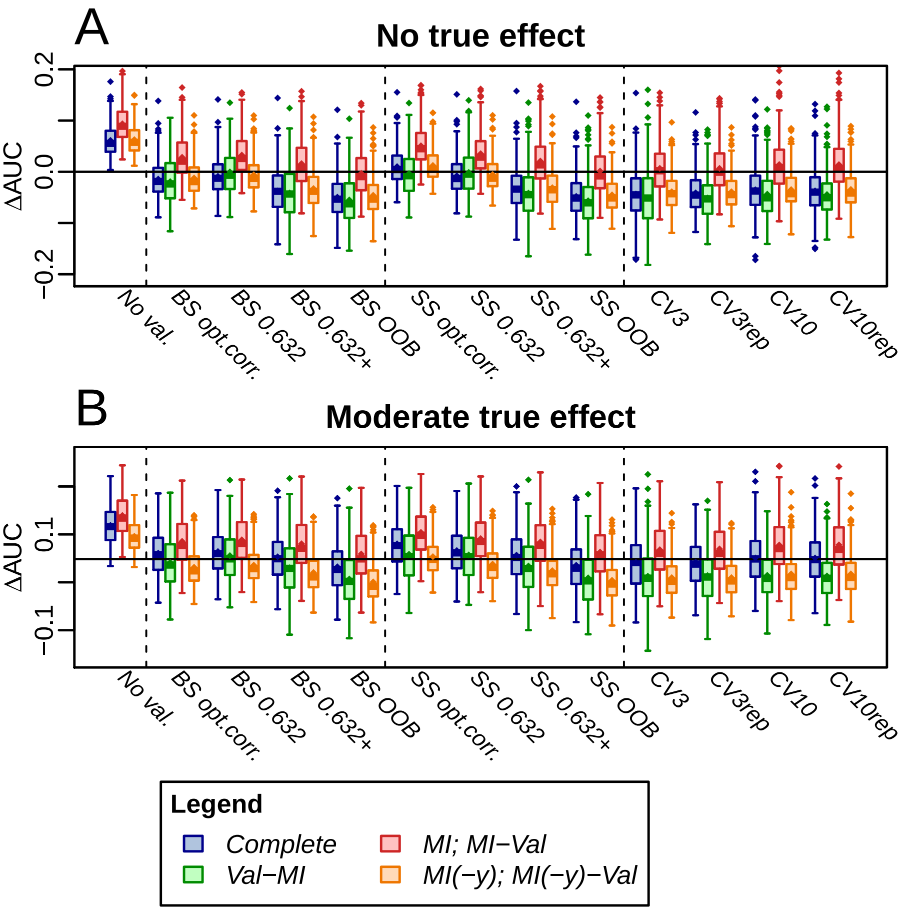

Figure S5: Simulation distribution of ΔAUC estimates obtained by different strategies. Boxplots showing distribution of ΔAUC estimates across the 250 simulated data sets in a setting with moderate sample size ($n = 200$), $p_0 = 1$ baseline covariate and $p_1 = 10$ additional covariates, moderate missing at random (MAR) missingness in additional covariates ($miss_1 = 25\%$ of values missing, while $miss_0 = 0\%$), balanced outcome class distribution ($frac = 0.5$) and uncorrelated covariates ($\rho_1 = 0$) in the absence (theoretical $\Delta auc = 0$; **A**) and presence (theoretical $\Delta auc = 0.08$; **B**) of a moderate true added effect of the covariates on the outcome. The horizontal line denotes 'true' ΔAUC related to a complete data set of size 200 (which is not necessarily equal to theoretical Δauc; see text). *BS*, bootstrap; *CVK(rep)*, (repeated) K-fold CV; *MI*, multiple imputation; *MI(-y)*, multiple imputation without including the outcome; *No val.*, no validation (i.e., apparent performance); *OOB*, out-of-bag estimate; *opt.corr.*, ordinary optimism-corrected estimate; *SS*, subsampling; *Val*, validation.

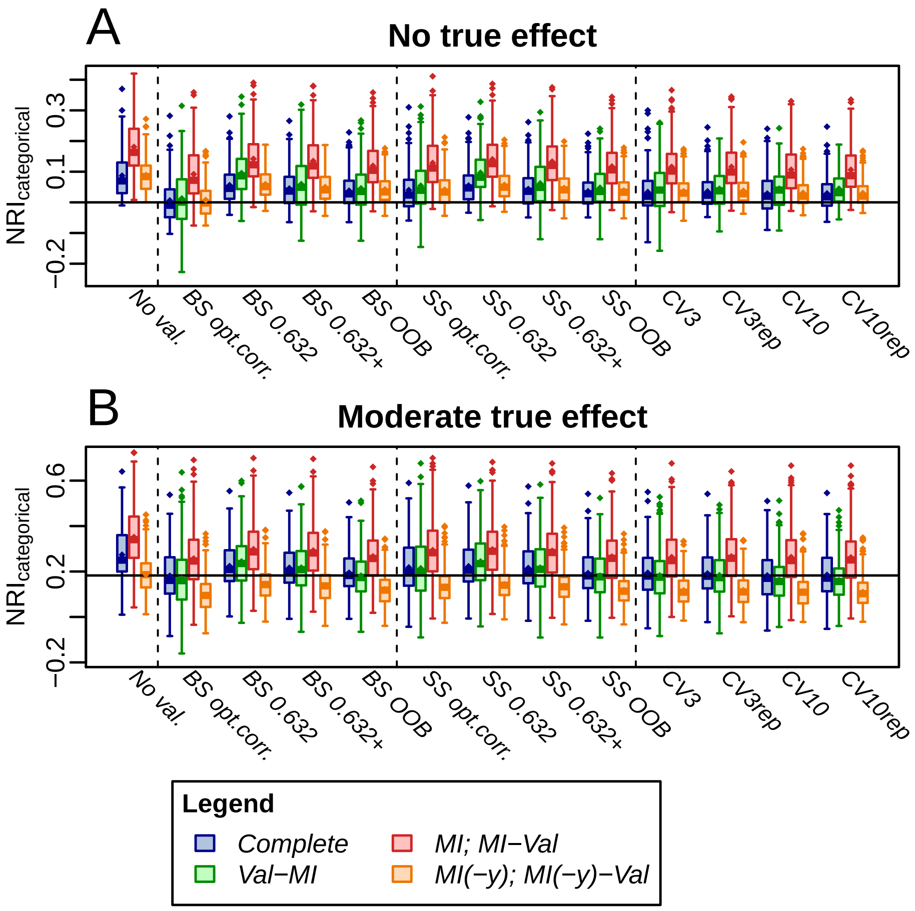

Figure S6: Simulation distribution of categorical NRI estimates obtained by different strategies. Boxplots showing distribution of categorical net reclassification improvement (NRI) estimates across the 250 simulated data sets in a setting with moderate sample size ($n = 200$), $p_0 = 1$ baseline covariate and $p_1 = 10$ additional covariates, moderate missing at random (MAR) missingness in additional covariates ($miss_1 = 25\%$ of values missing, while $miss_0 = 0\%$), balanced outcome class distribution ($frac = 0.5$) and uncorrelated covariates ($\rho_1 = 0$) in the absence (theoretical $\Delta auc = 0$; **A**) and presence (theoretical $\Delta auc = 0.08$; **B**) of a moderate true added effect of the covariates on the outcome. The horizontal line denotes 'true' categorical NRI related to a complete data set of size 200 (see text). *BS*, bootstrap; *CVK(rep)*, (repeated) K-fold CV; *MI*, multiple imputation; *MI(-y)*, multiple imputation without including the outcome; *No val.*, no validation (i.e., apparent performance); *OOB*, out-of-bag estimate; *opt.corr.*, ordinary optimism-corrected estimate; *SS*, subsampling; *Val*, validation.

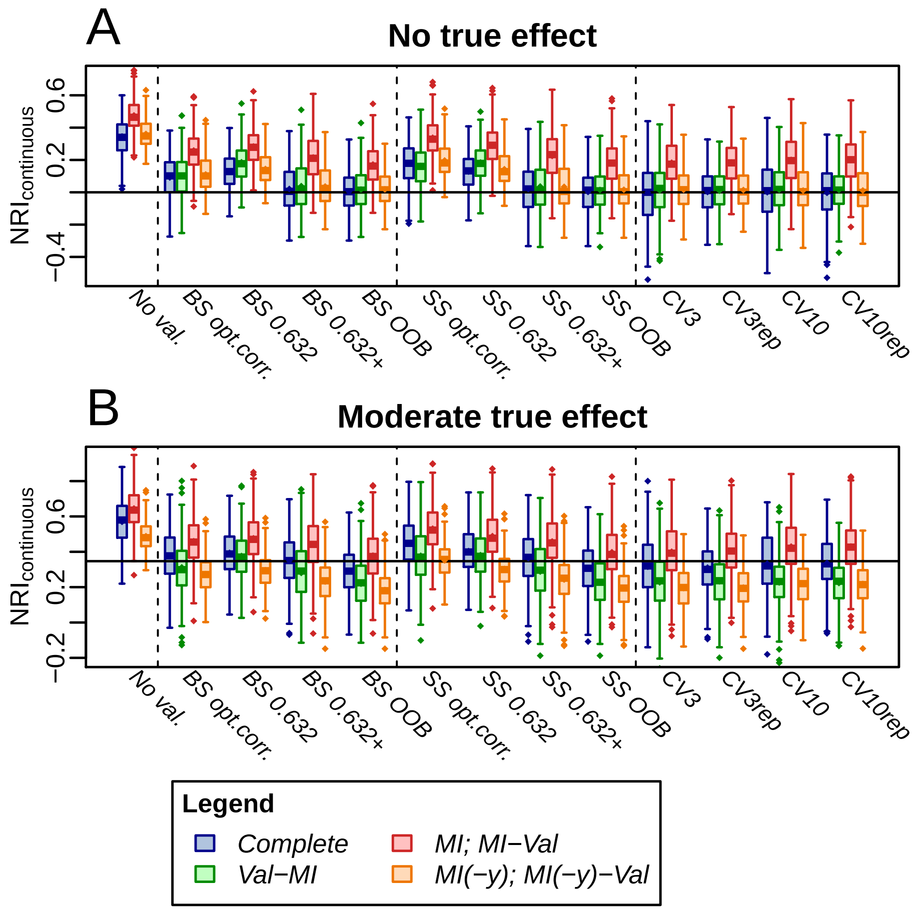

Figure S7: Simulation distribution of continuous NRI estimates obtained by different strategies. Boxplots showing distribution of continuous net reclassification improvement (NRI) estimates across the 250 simulated data sets in a setting with moderate sample size ($n = 200$), $p_0 = 1$ baseline covariate and $p_1 = 10$ additional covariates, moderate missing at random (MAR) missingness in additional covariates ($miss_1 = 25\%$ of values missing, while $miss_0 = 0\%$), balanced outcome class distribution ($frac = 0.5$) and uncorrelated covariates ($\rho_1 = 0$) in the absence (theoretical $\Delta auc = 0$; **A**) and presence (theoretical $\Delta auc = 0.08$; **B**) of a moderate true added effect of the covariates on the outcome. The horizontal line denotes 'true' continuous NRI related to a complete data set of size 200 (see text). *BS*, bootstrap; *CVK(rep)*, (repeated) K-fold CV; *MI*, multiple imputation; *MI(-y)*, multiple imputation without including the outcome; *No val.*, no validation (i.e., apparent performance); *OOB*, out-of-bag estimate; *opt.corr.*, ordinary optimism-corrected estimate; *SS*, subsampling; *Val*, validation.

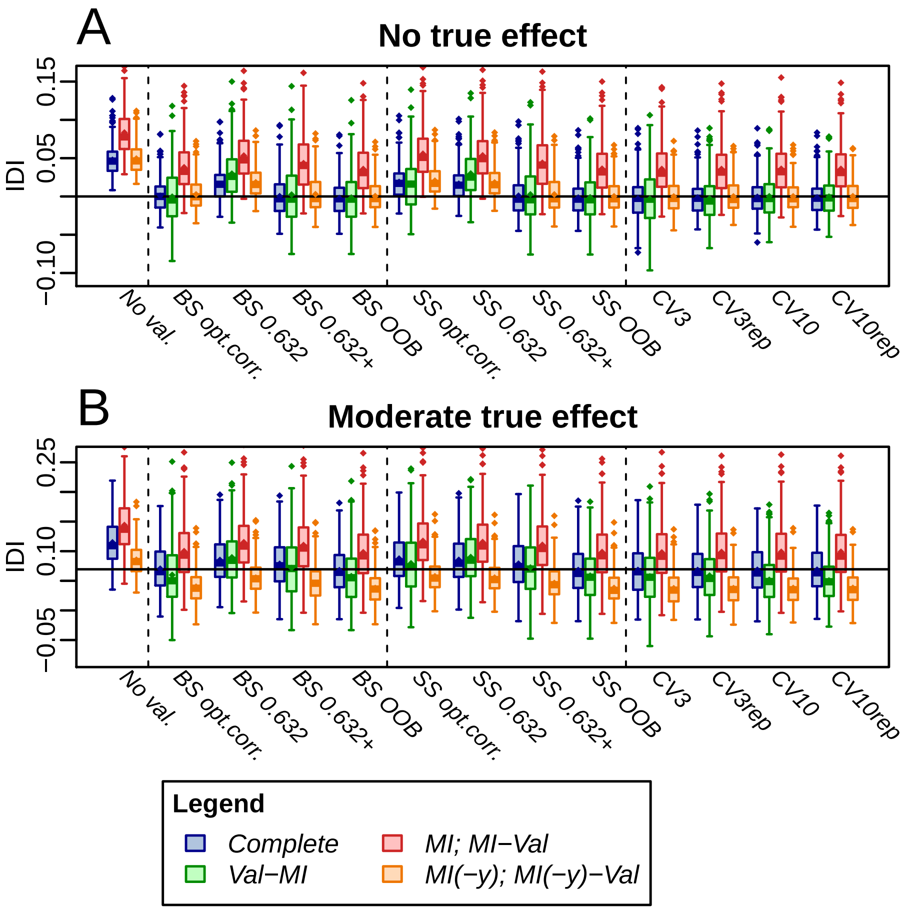

Figure S8: Simulation distribution of IDI estimates obtained by different strategies. Boxplots showing distribution of integrated discrimination improvement (IDI) estimates across the 250 simulated data sets in a setting with moderate sample size ($n = 200$), $p_0 = 1$ baseline covariate and $p_1 = 10$ additional covariates, moderate missing at random (MAR) missingness in additional covariates ($miss_1 = 25\%$ of values missing, while $miss_0 = 0\%$), balanced outcome class distribution ($frac = 0.5$) and uncorrelated covariates ($\rho_1 = 0$) in the absence (theoretical $\Delta auc = 0$; **A**) and presence (theoretical $\Delta auc = 0.08$; **B**) of a moderate true added effect of the covariates on the outcome. The horizontal line denotes 'true' IDI related to a complete data set of size 200 (see text). *BS*, bootstrap; *CVK(rep)*, (repeated) K-fold CV; *MI*, multiple imputation; *MI(-y)*, multiple imputation without including the outcome; *No val.*, no validation (i.e., apparent performance); *OOB*, out-of-bag estimate; *opt.corr.*, ordinary optimism-corrected estimate; *SS*, subsampling; *Val*, validation.

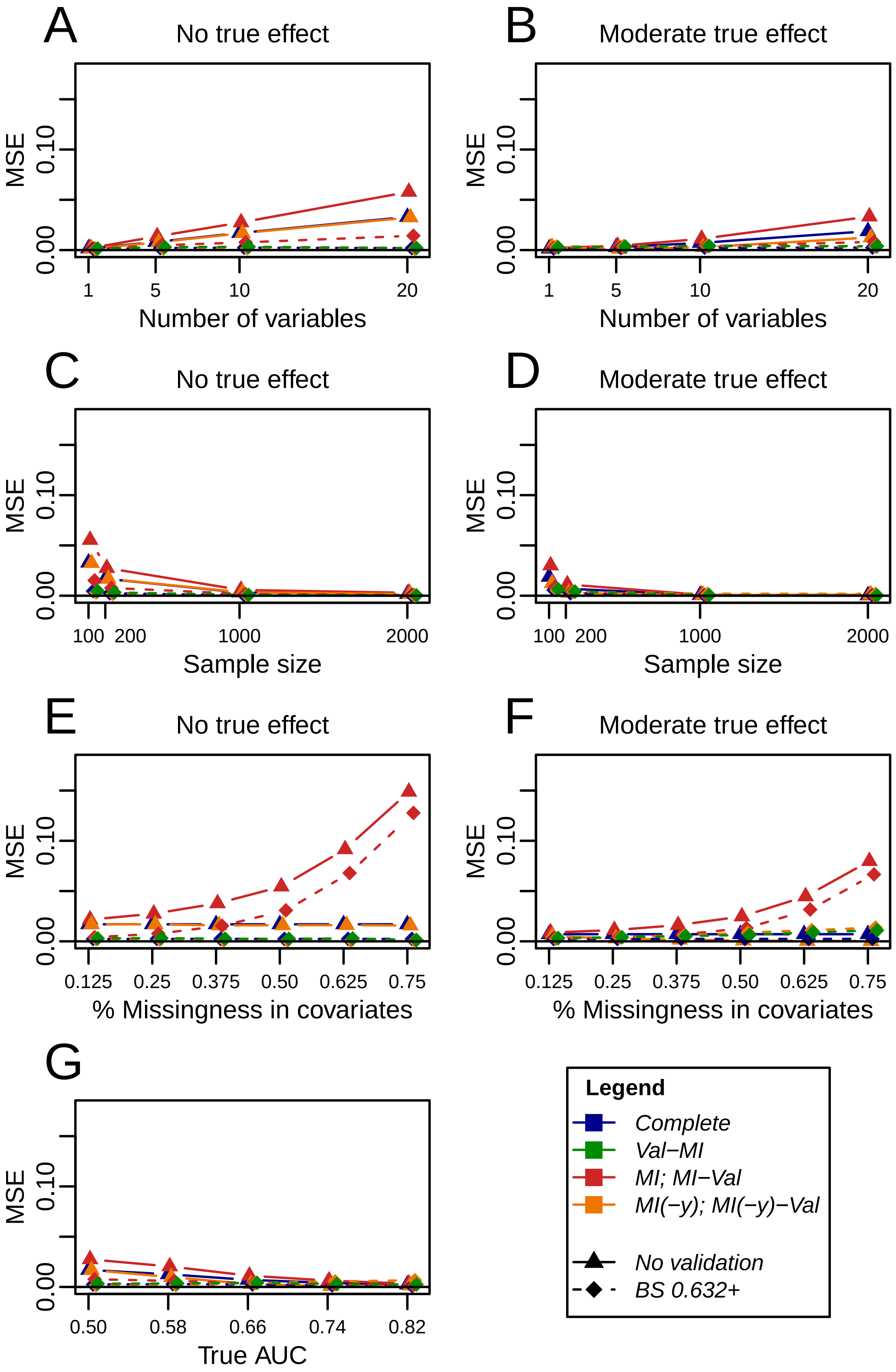

Figure S9: Mean squared error of AUC estimates obtained by different strategies based on bootstrapping. Mean squared error (MSE) is shown for one varying data set characteristic in each panel (**A,B** number of covariates p; **C,D** sample size n; **E,F** degree of missingness $miss$; **G** true effect auc), while keeping all remaining characteristics constant: sample size ($n = 200$), $p = 10$ covariates, 25% missing values, missing at random (MAR) missiness, balanced outcome class distribution ($frac = 0.5$), uncorrelated covariates ($\rho = 0$). Results are shown for absence (theoretical $auc = 0.5$; **A,C,E,G**) and presence (theoretical $auc = 0.66$; **B,D,F,G**) of a moderate true effect of the covariates on the outcome.

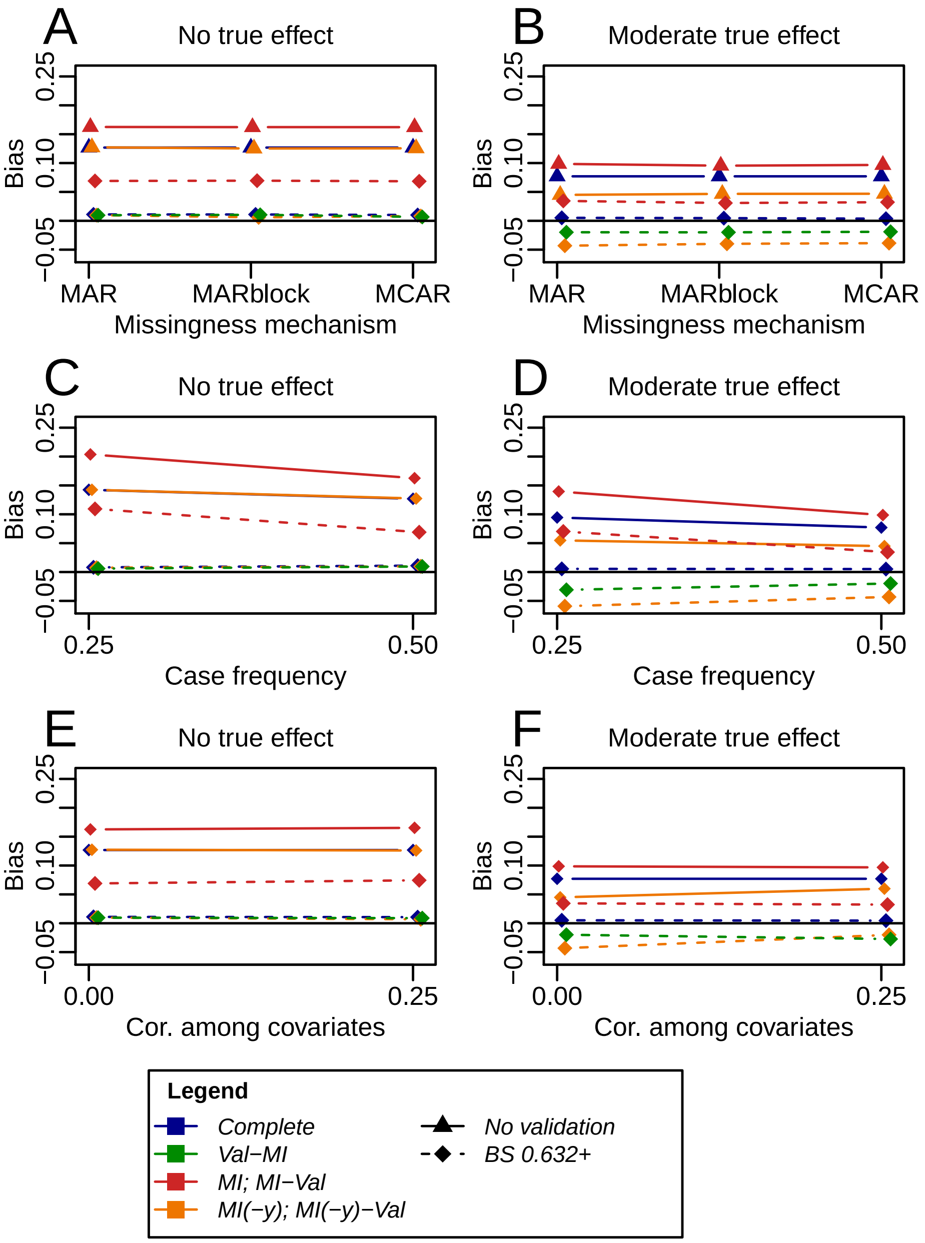

Figure S10: Bias of AUC estimates obtained by different strategies based on bootstrapping – Influence of further data characteristics. Bias is shown for one varying data set characteristic in each panel (**A,B** missingness mechanism; **C,D** outcome class frequency *frac*; **E,F** correlation among the covariates ρ), while keeping all remaining characteristics constant: sample size ($n = 200$), $p = 10$ covariates, 25% missing values, missing at random (MAR) missingness, balanced outcome class distribution ($frac = 0.5$), uncorrelated covariates ($\rho = 0$). Results are shown for absence (theoretical $auc = 0.5$; **A,C,E**) and presence (theoretical $auc = 0.66$; **B,D,F**) of a moderate true effect of the covariates on the outcome.

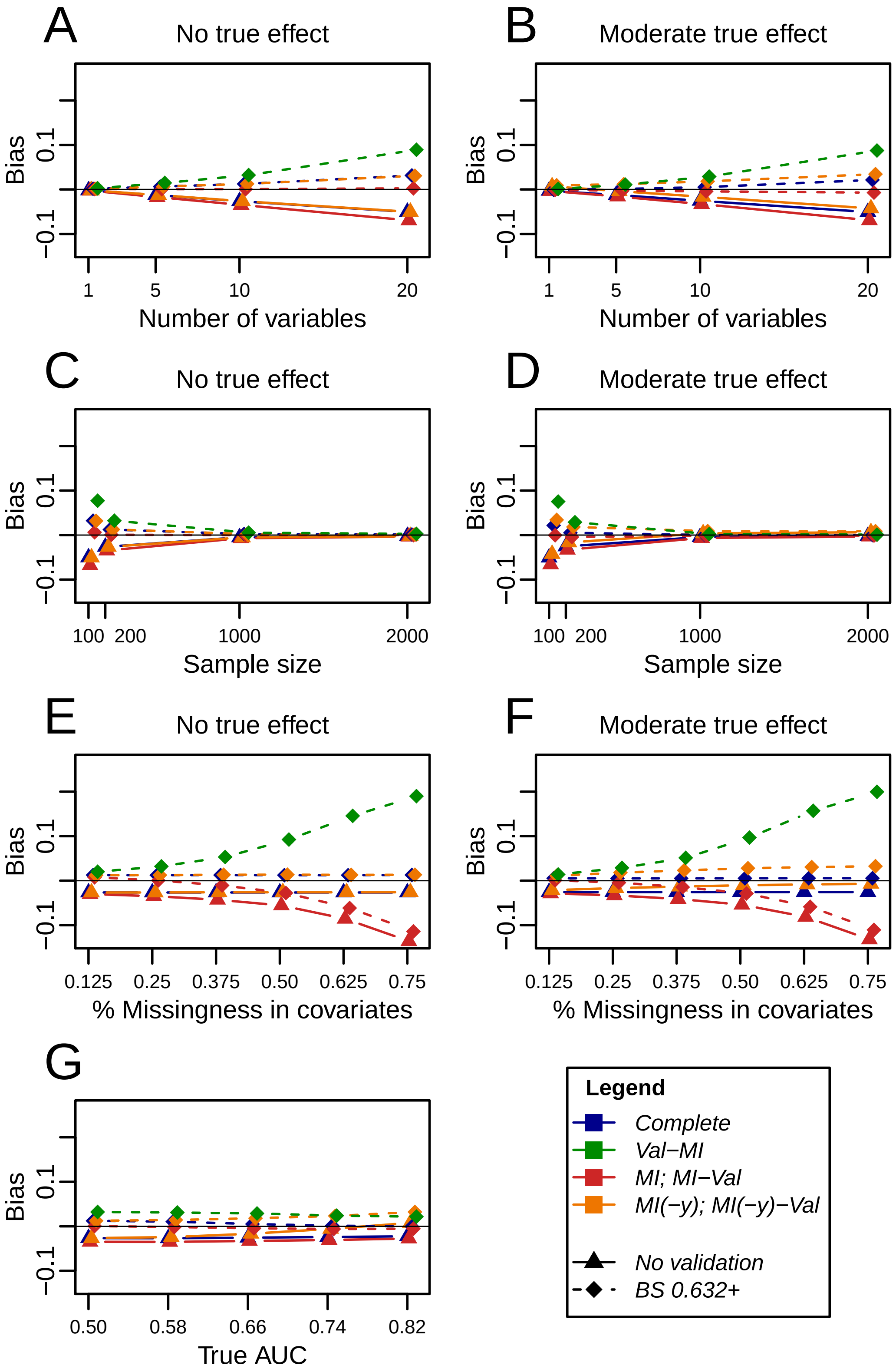

Figure S11: Bias of Brier score estimates obtained by different strategies based on bootstrapping. Bias is shown for one varying data set characteristic in each panel (**A,B** number of covariates p; **C,D** sample size n; **E,F** degree of missingness $miss$; **G** true effect auc), while keeping all remaining characteristics constant: sample size ($n = 200$), $p = 10$ covariates, 25% missing values, missing at random (MAR) missingness, balanced outcome class distribution ($frac = 0.5$), uncorrelated covariates ($\rho = 0$). Results are shown for absence (theoretical $auc = 0.5$; **A,C,E,G**) and presence (theoretical $auc = 0.66$; **B,D,F,G**) of a moderate true effect of the covariates on the outcome.

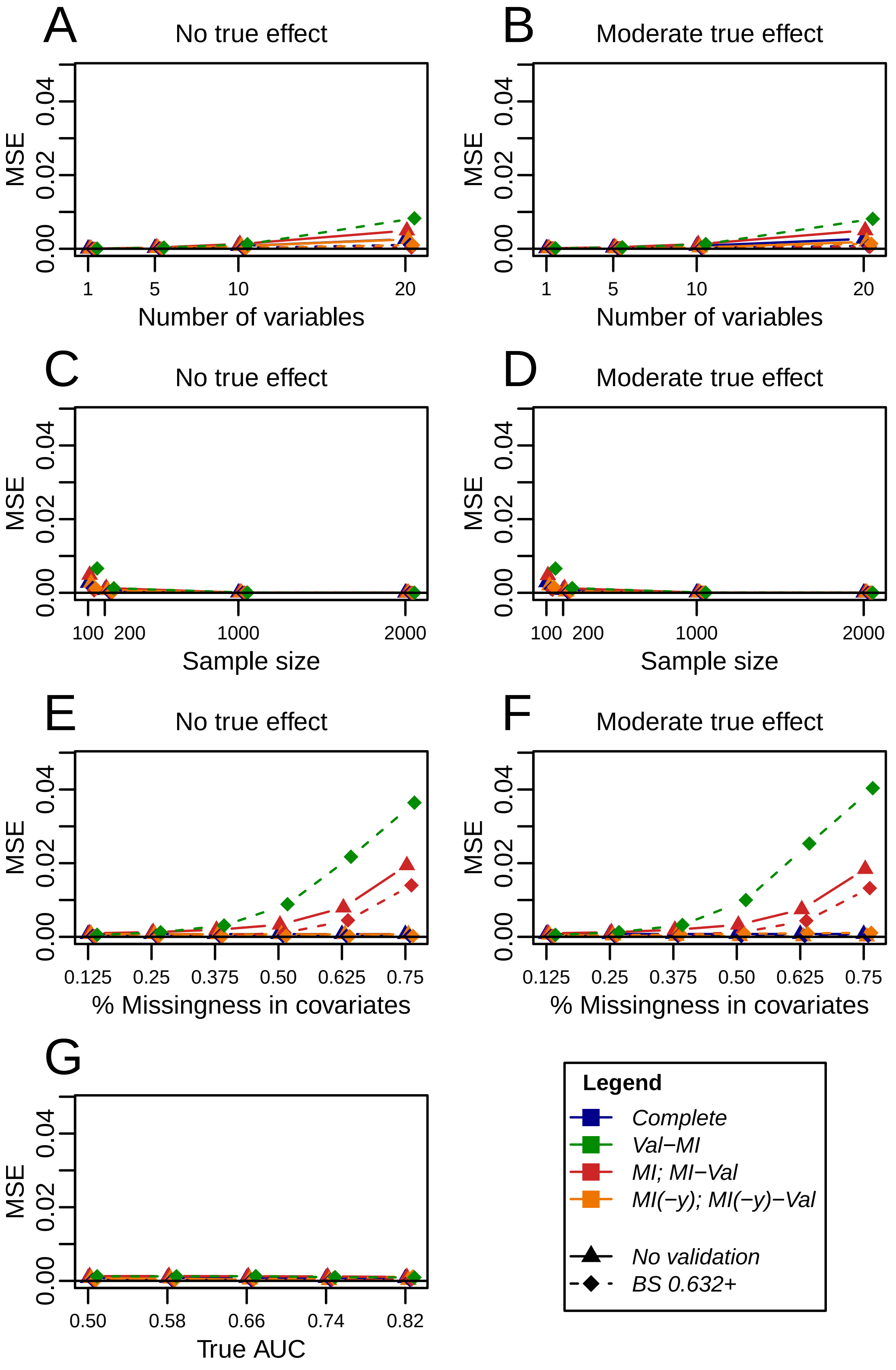

Figure S12: Mean squared error of Brier score estimates obtained by different strategies based on bootstrapping. Mean squared error (MSE) is shown for one varying data set characteristic in each panel (**A,B** number of covariates p; **C,D** sample size n; **E,F** degree of missingness $miss$; **G** true effect auc), while keeping all remaining characteristics constant: sample size ($n = 200$), $p = 10$ covariates, 25% missing values, missing at random (MAR) missingness, balanced outcome class distribution ($frac = 0.5$), uncorrelated covariates ($\rho = 0$). Results are shown for absence (theoretical $auc = 0.5$; **A,C,E,G**) and presence (theoretical $auc = 0.66$; **B,D,F,G**) of a moderate true effect of the covariates on the outcome.

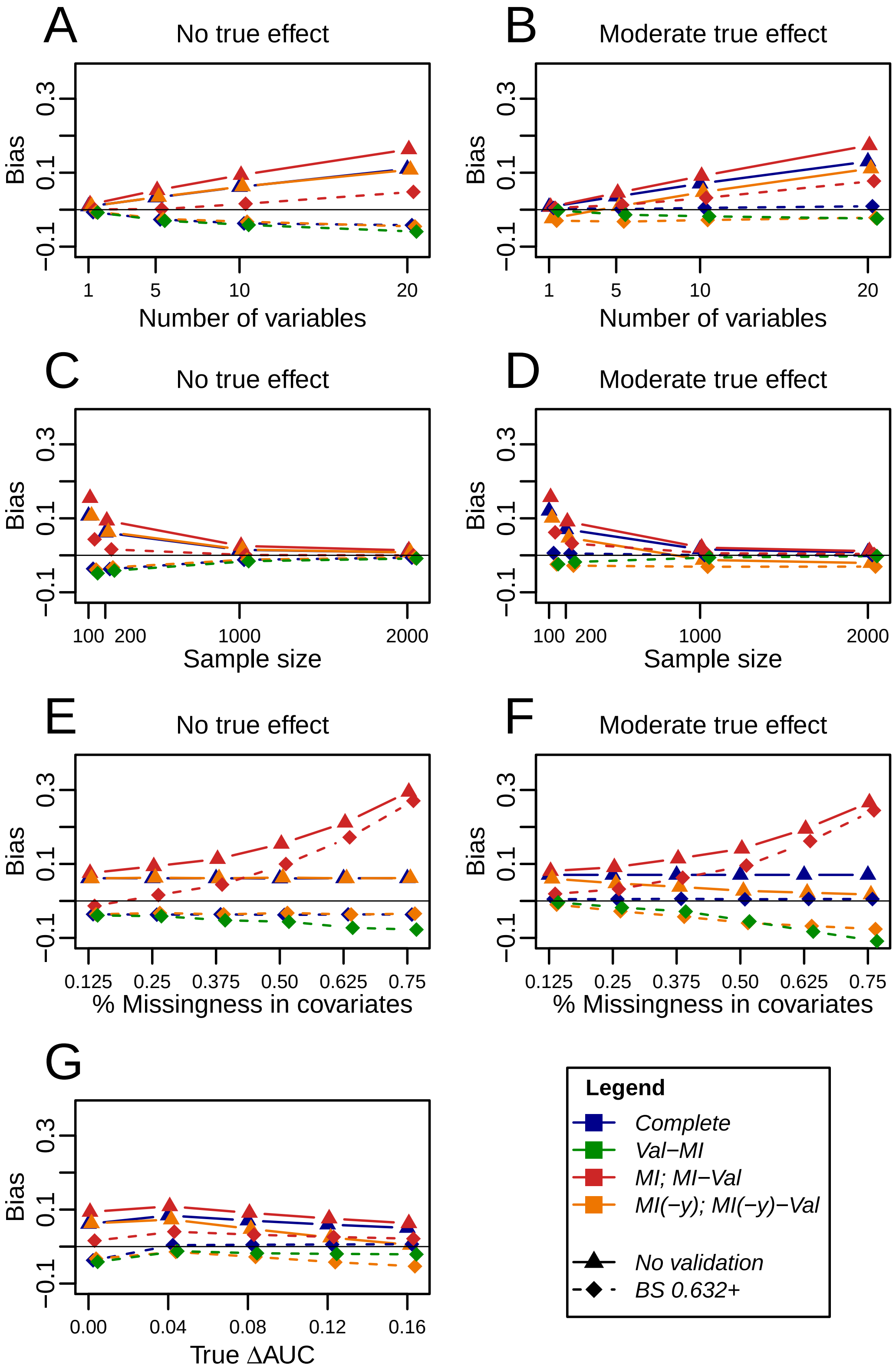

Figure S13: Bias of ΔAUC estimates obtained by different strategies based on bootstrapping. Bias is shown for one varying data set characteristic in each panel (**A,B** number of covariates p; **C,D** sample size n; **E,F** degree of missingness *miss*; **G** true effect Δauc), while keeping all remaining characteristics constant: sample size ($n = 200$), $p = 10$ covariates, 25% missing values, missing at random (MAR) missingness, balanced outcome class distribution ($frac = 0.5$) and uncorrelated covariates ($\rho = 0$). Results are shown for absence (theoretical $\Delta auc = 0$; **A,C,E,G**) and presence (theoretical $\Delta auc = 0.08$; **B,D,F,G**) of a moderate true added effect of the covariates on the outcome.

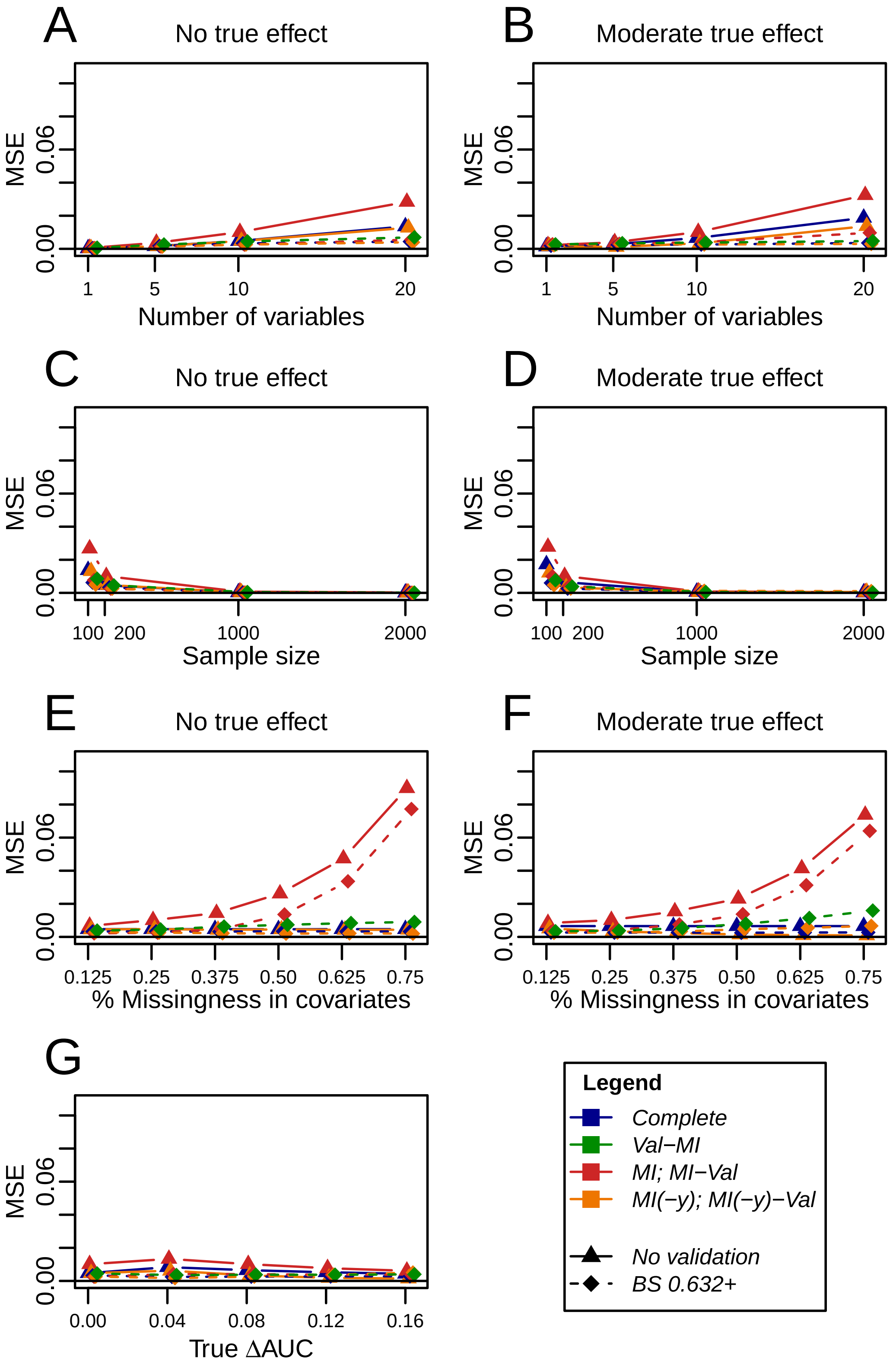

Figure S14: Mean squared error of ΔAUC estimates obtained by different strategies based on bootstrapping. Mean squared error (MSE) is shown for one varying data set characteristic in each panel (**A,B** number of covariates p; **C,D** sample size n; **E,F** degree of missingness $miss$; **G** true effect Δauc), while keeping all remaining characteristics constant: sample size ($n = 200$), $p = 10$ covariates, 25% missing values, missing at random (MAR) missingness, balanced outcome class distribution ($frac = 0.5$) and uncorrelated covariates ($\rho = 0$). Results are shown for absence (theoretical $\Delta auc = 0$; **A,C,E,G**) and presence (theoretical $\Delta auc = 0.08$; **B,D,F,G**) of a moderate true added effect of the covariates on the outcome.

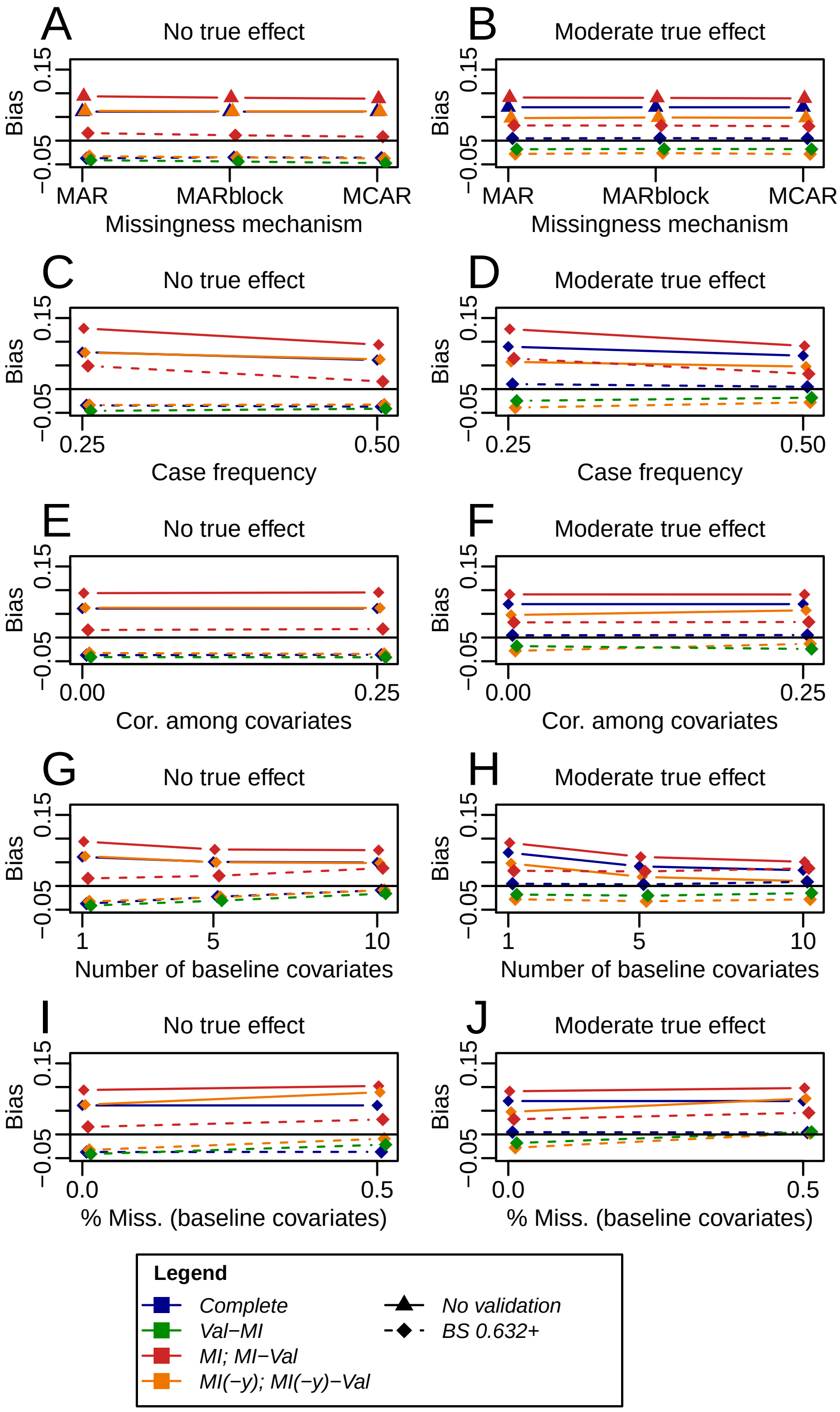

Figure S15: Bias of ΔAUC estimates obtained by different strategies based on bootstrapping – Influence of further data characteristics. Bias is shown for one varying data set characteristic in each panel (**A,B** missingness mechanism; **C,D** outcome class frequency *frac*; **E,F** correlation among the covariates ρ; **G,H** number of baseline covariates p_0 and **I,J** proportion of missing values among the baseline covariates), while keeping all remaining characteristics constant: sample size ($n = 200$), $p_0 = 1$ baseline covariate and $p = 10$ additional covariates, $miss_0 = 0$ and $miss = 25\%$ missing values in baseline and additional covariates, respectively, missing at random (MAR) missingness, balanced outcome class distribution ($frac = 0.5$) and uncorrelated covariates ($\rho = 0$). Results are shown for absence (theoretical $\Delta auc = 0$; **A,C,E,G,I**) and presence (theoretical $\Delta auc = 0.08$; **B,D,F,H,J**) of a moderate true added effect of the covariates on the outcome.

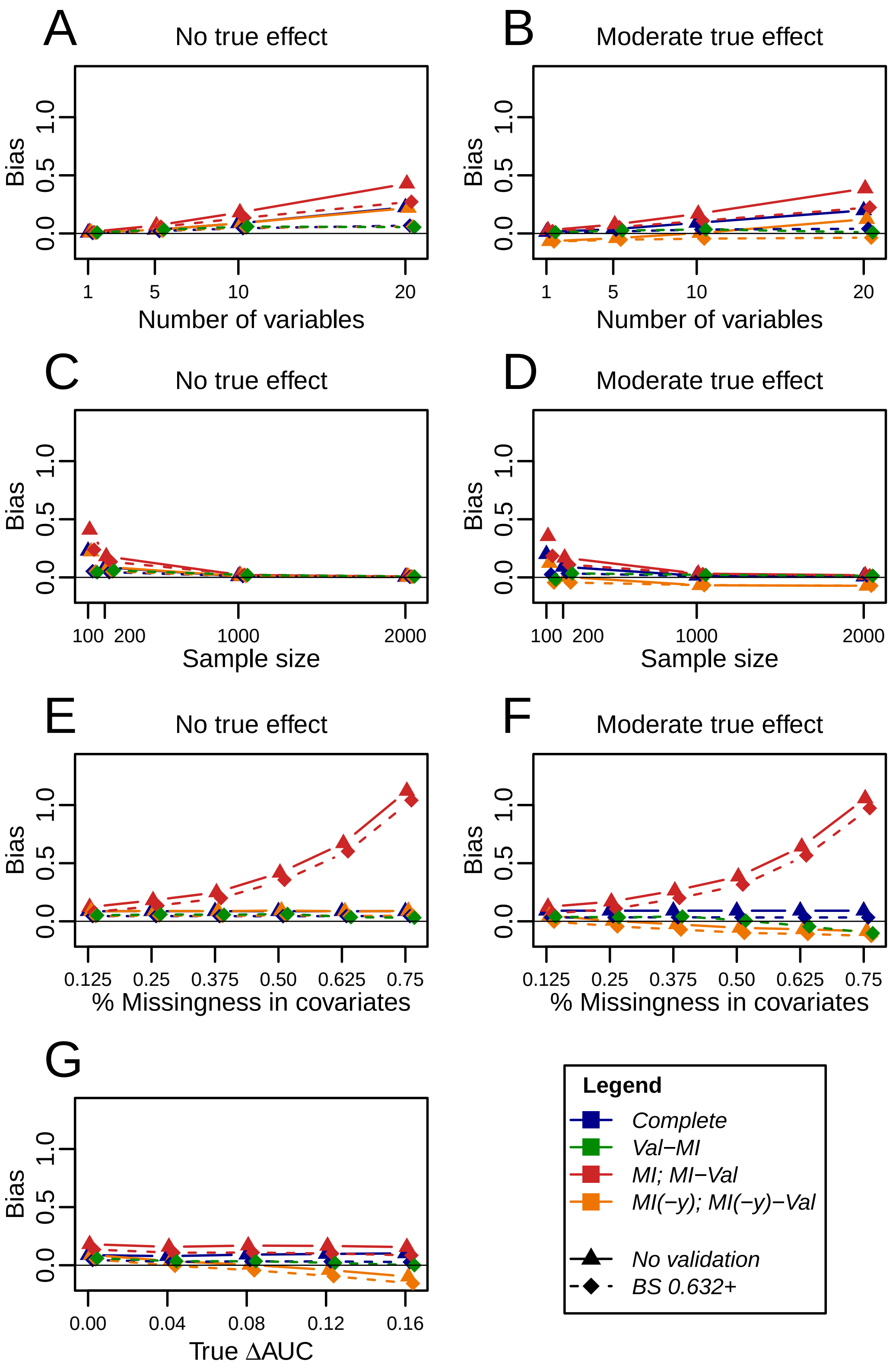

Figure S16: Bias of categorical net reclassification improvement (NRI) estimates obtained by different strategies based on bootstrapping. Bias is shown for one varying data set characteristic in each panel (**A,B** number of covariates p; **C,D** sample size n; **E,F** degree of missingness $miss$; **G** true effect Δauc), while keeping all remaining characteristics constant: sample size ($n = 200$), $p_0 = 1$ baseline covariate and $p_1 = 10$ additional covariates, $miss_0 = 0$ and $miss_1 = 25\%$ missing values in baseline and additional covariates, respectively, missing at random (MAR) missingness, balanced outcome class distribution ($frac = 0.5$) and uncorrelated covariates ($\rho_1 = 0$). Results are shown for absence (theoretical $\Delta auc = 0$; **A,C,E,G**) and presence (theoretical $\Delta auc = 0.08$; **B,D,F,G**) of a moderate true added effect of the covariates on the outcome.

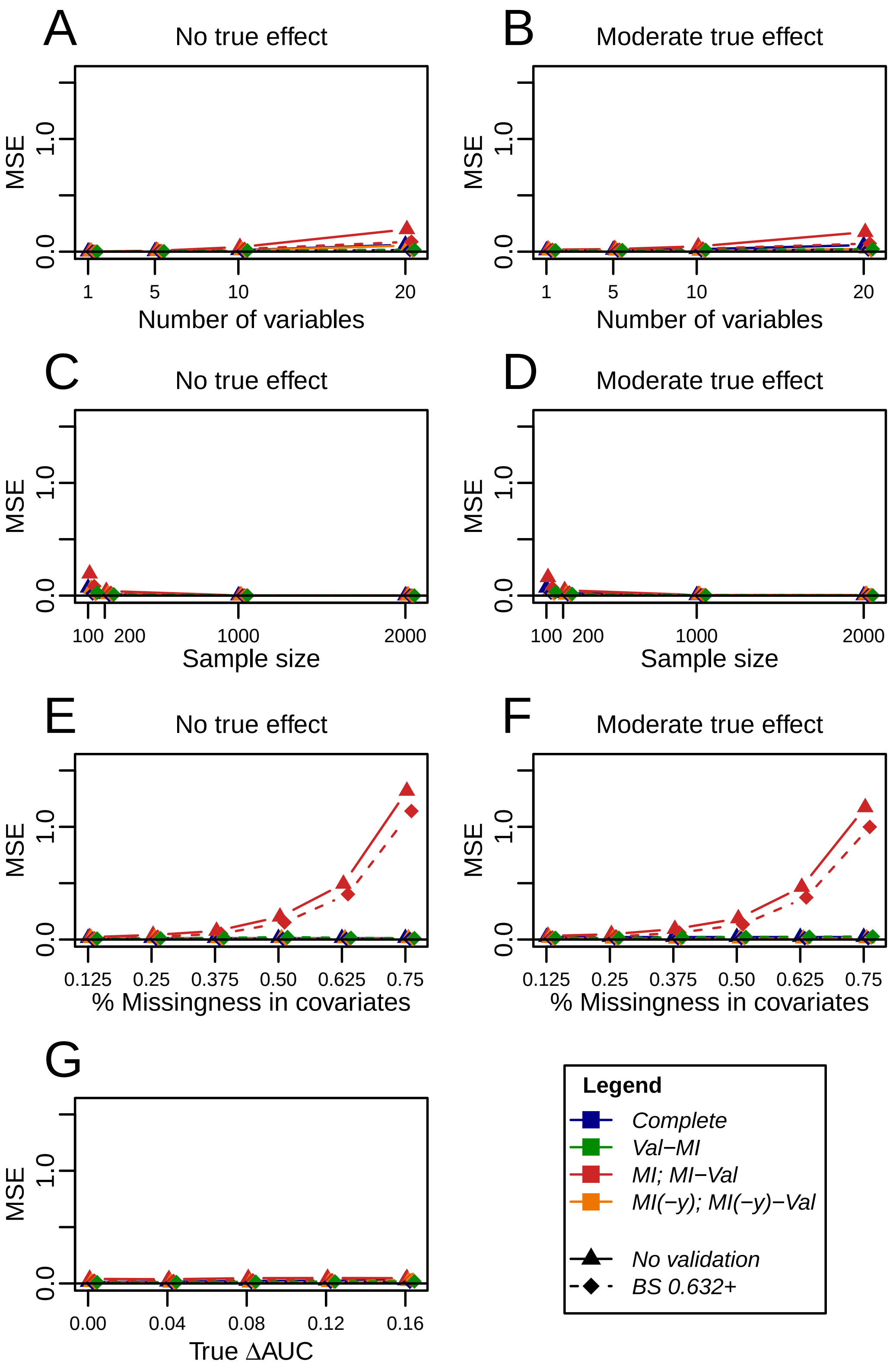

Figure S17: Mean squared error of categorical net reclassification improvement (NRI) estimates obtained by different strategies based on bootstrapping. Mean squared error (MSE) is shown for one varying data set characteristic in each panel (**A,B** number of covariates p; **C,D** sample size n; **E,F** degree of missingness $miss$; **G** true effect Δauc), while keeping all remaining characteristics constant: sample size ($n = 200$), $p_0 = 1$ baseline covariate and $p_1 = 10$ additional covariates, $miss_0 = 0$ and $miss_1 = 25\%$ missing values in baseline and additional covariates, respectively, missing at random (MAR) missingness, balanced outcome class distribution ($frac = 0.5$) and uncorrelated covariates ($\rho_1 = 0$). Results are shown for absence (theoretical $\Delta auc = 0$; **A,C,E,G**) and presence (theoretical $\Delta auc = 0.08$; **B,D,F,G**) of a moderate true added effect of the covariates on the outcome.

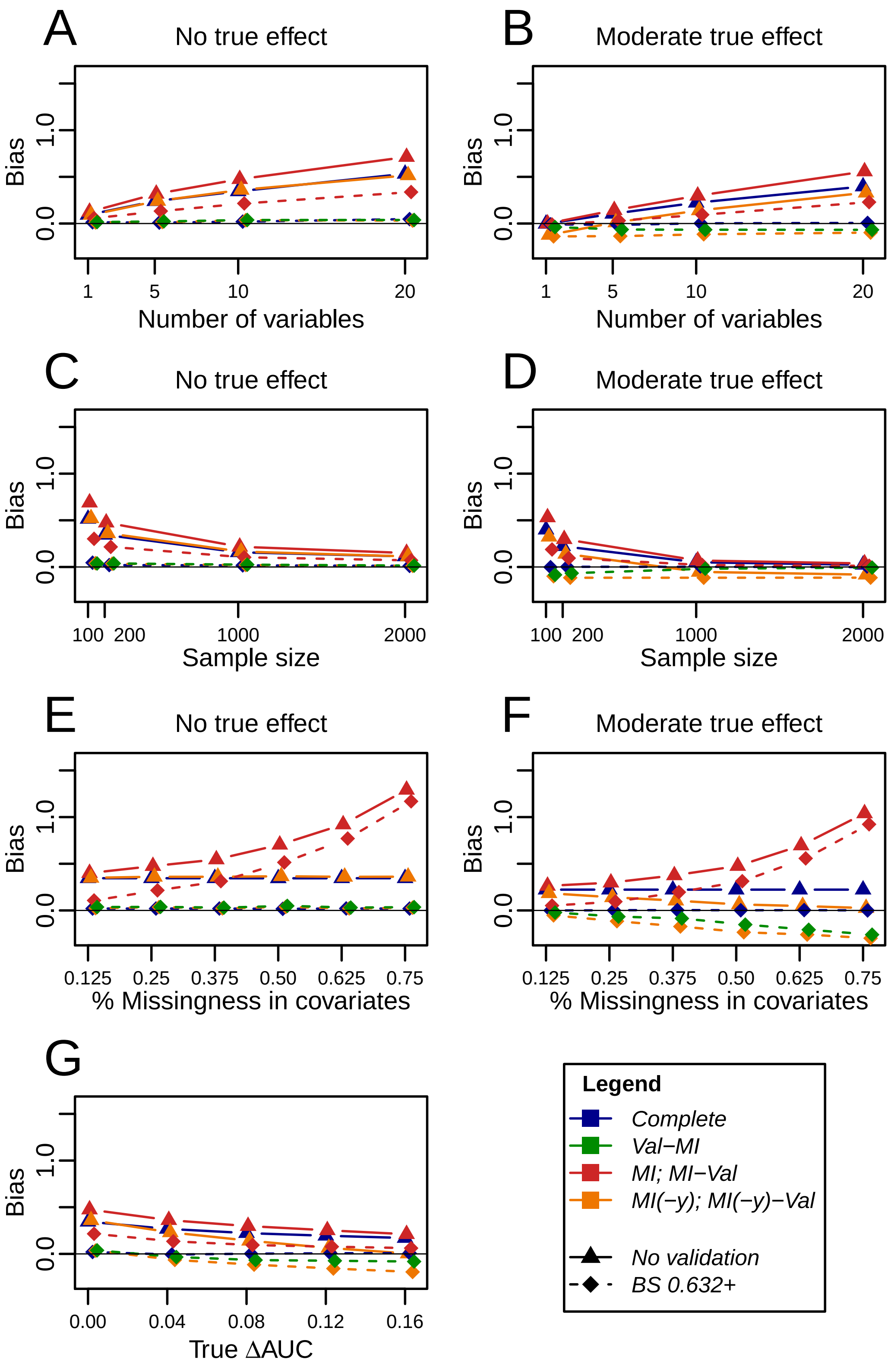

Figure S18: Bias of continuous net reclassification improvement (NRI) estimates obtained by different strategies based on bootstrapping. Bias is shown for one varying data set characteristic in each panel (**A,B** number of covariates p; **C,D** sample size n; **E,F** degree of missingness $miss$; **G** true effect Δauc), while keeping all remaining characteristics constant: sample size ($n = 200$), $p_0 = 1$ baseline covariate and $p_1 = 10$ additional covariates, $miss_0 = 0$ and $miss_1 = 25\%$ missing values in baseline and additional covariates, respectively, missing at random (MAR) missingness, balanced outcome class distribution ($frac = 0.5$) and uncorrelated covariates ($\rho_1 = 0$). Results are shown for absence (theoretical $\Delta auc = 0$; **A,C,E,G**) and presence (theoretical $\Delta auc = 0.08$; **B,D,F,G**) of a moderate true added effect of the covariates on the outcome.

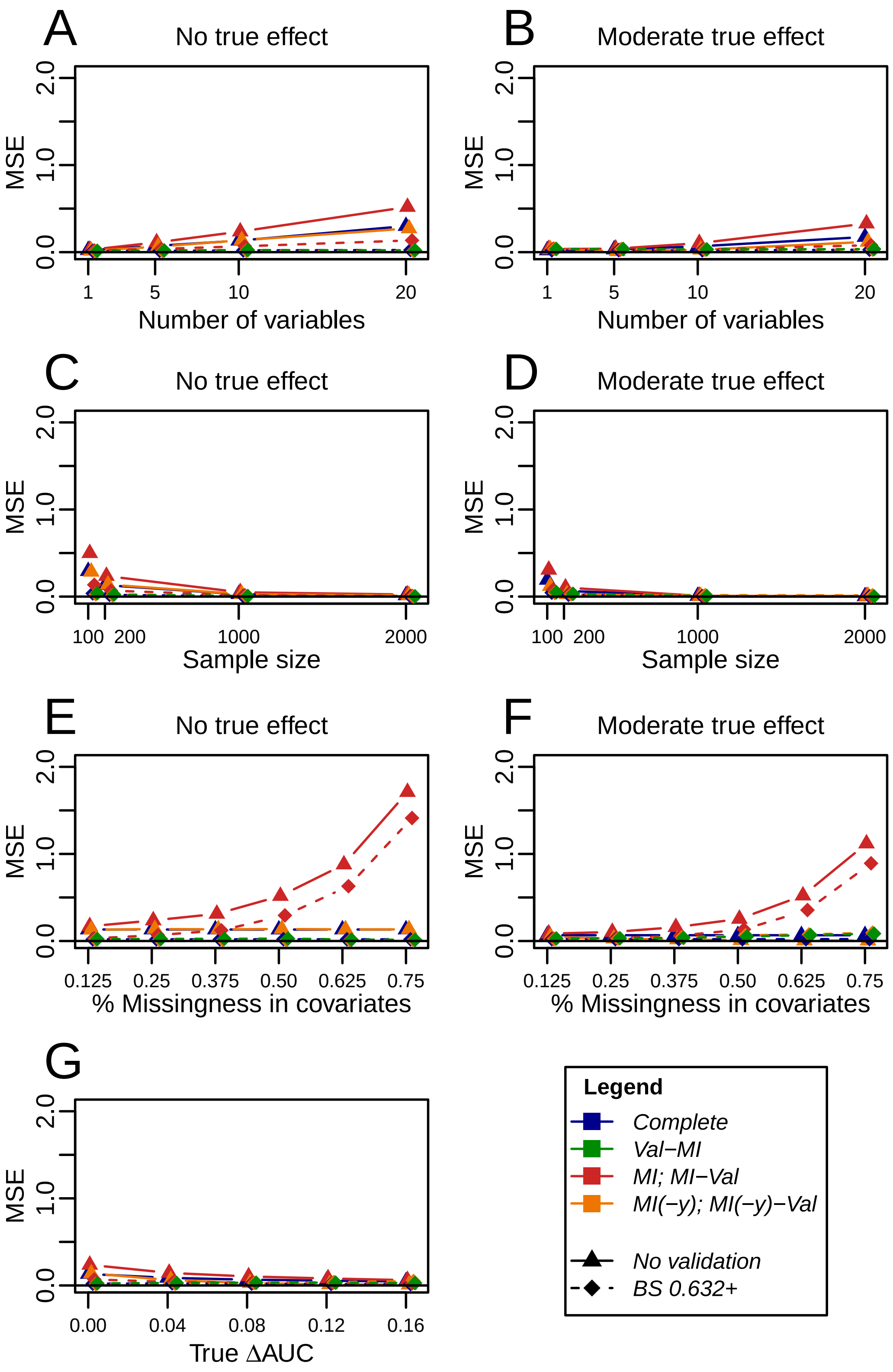

Figure S19: Mean squared error of continuous net reclassification improvement (NRI) estimates obtained by different strategies based on bootstrapping. Mean squared error (MSE) is shown for one varying data set characteristic in each panel (**A,B** number of covariates p; **C,D** sample size n; **E,F** degree of missingness $miss$; **G** true effect Δauc), while keeping all remaining characteristics constant: sample size ($n = 200$), $p_0 = 1$ baseline covariate and $p_1 = 10$ additional covariates, $miss_0 = 0$ and $miss_1 = 25\%$ missing values in baseline and additional covariates, respectively, missing at random (MAR) missingness, balanced outcome class distribution ($frac = 0.5$) and uncorrelated covariates ($\rho_1 = 0$). Results are shown for absence (theoretical $\Delta auc = 0$; **A,C,E,G**) and presence (theoretical $\Delta auc = 0.08$; **B,D,F,G**) of a moderate true added effect of the covariates on the outcome.

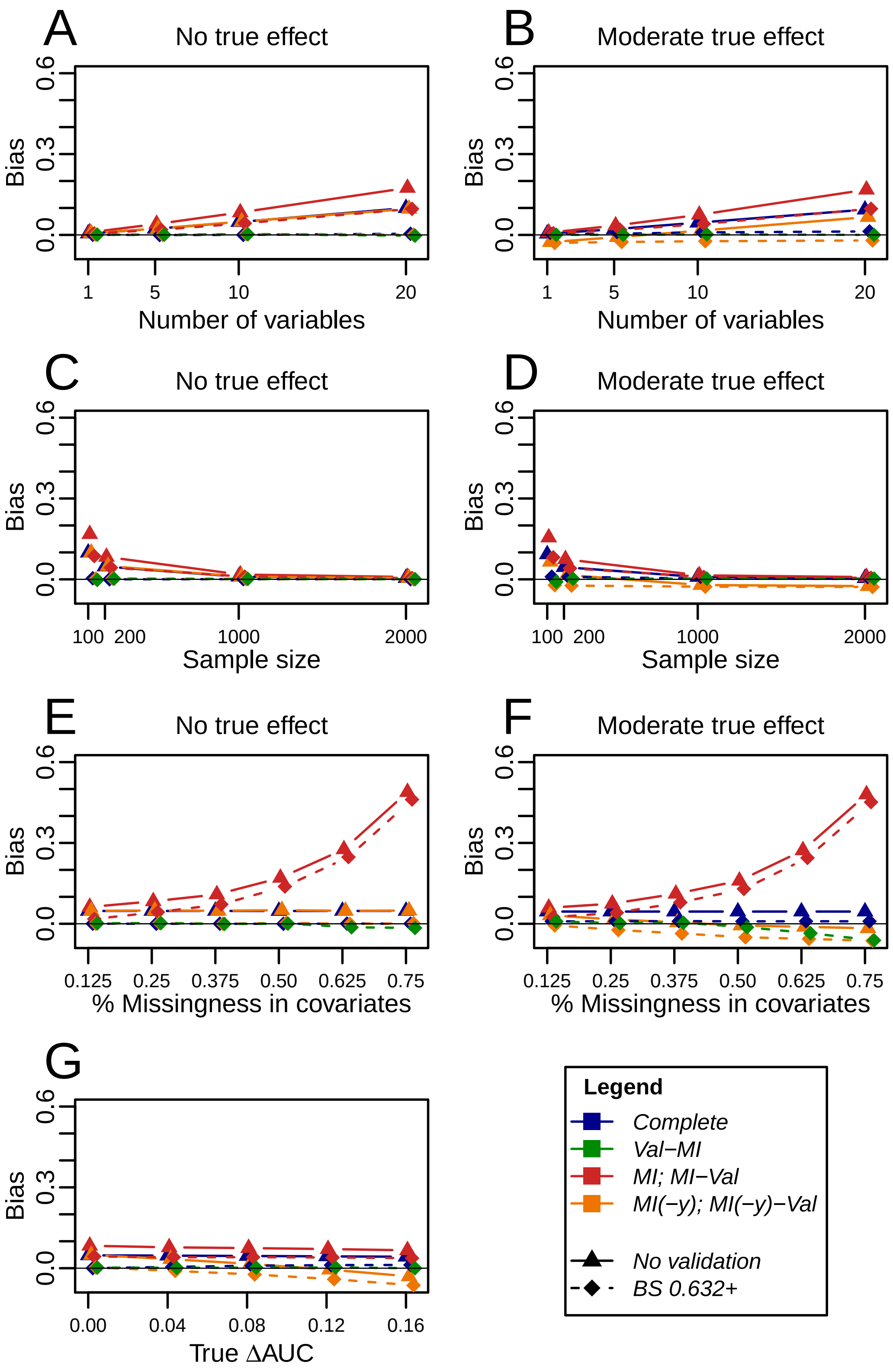

Figure S20: Bias of continuous integrated discrimination improvement (IDI) estimates obtained by different strategies based on bootstrapping. Bias is shown for one varying data set characteristic in each panel (**A,B** number of covariates p; **C,D** sample size n; **E,F** degree of missingness $miss$; **G** true effect Δauc), while keeping all remaining characteristics constant: sample size ($n = 200$), $p_0 = 1$ baseline covariate and $p_1 = 10$ additional covariates, $miss_0 = 0$ and $miss_1 = 25\%$ missing values in baseline and additional covariates, respectively, missing at random (MAR) missingness, balanced outcome class distribution ($frac = 0.5$) and uncorrelated covariates ($\rho_1 = 0$). Results are shown for absence (theoretical $\Delta auc = 0$; **A,C,E,G**) and presence (theoretical $\Delta auc = 0.08$; **B,D,F,G**) of a moderate true added effect of the covariates on the outcome.

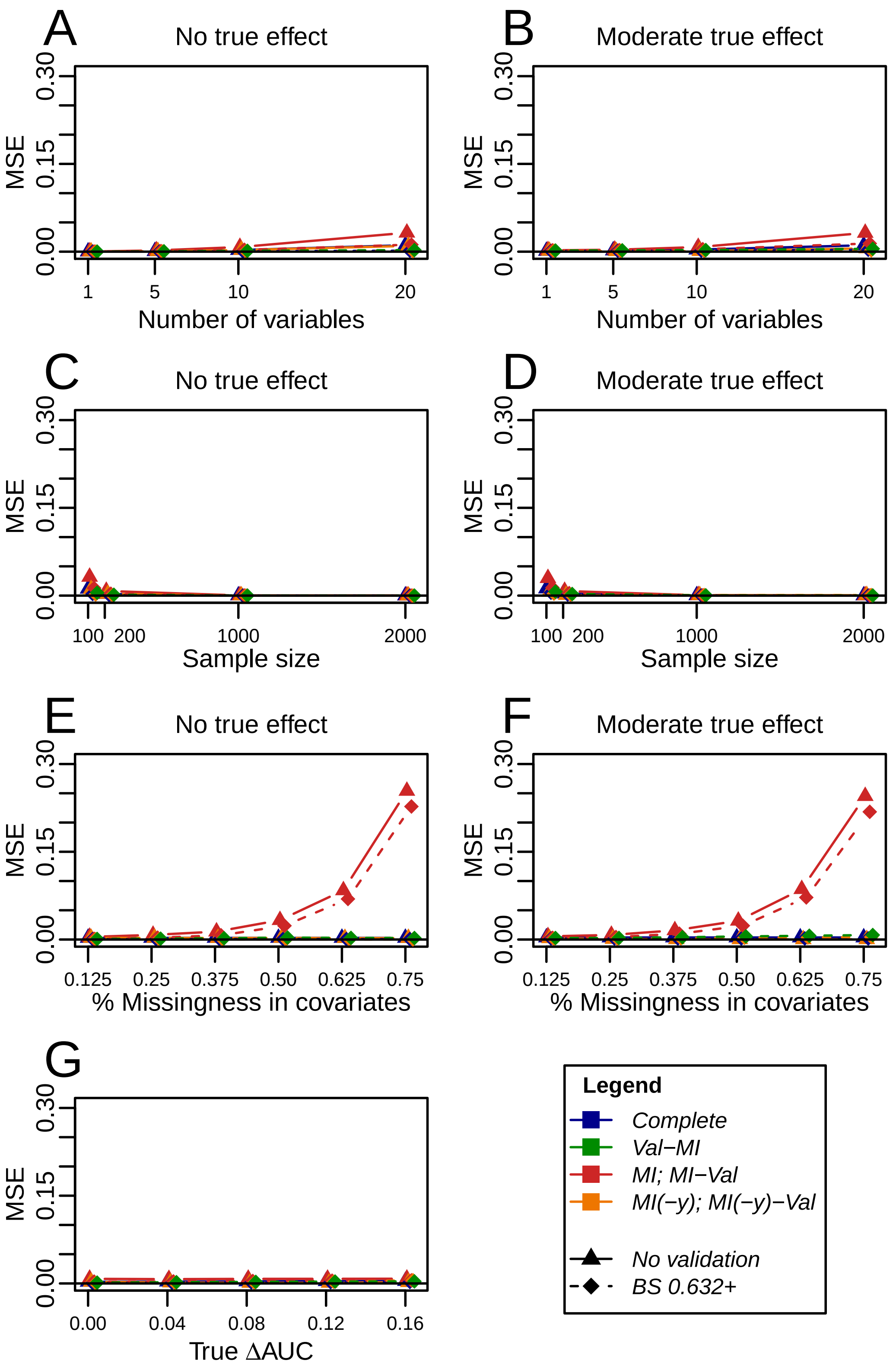

Figure S21: Mean squared error of integrated discrimination improvement (IDI) estimates obtained by different strategies based on bootstrapping. Mean squared error (MSE) is shown for one varying data set characteristic in each panel (**A,B** number of covariates p; **C,D** sample size n; **E,F** degree of missingness $miss$; **G** true effect Δauc), while keeping all remaining characteristics constant: sample size ($n = 200$), $p_0 = 1$ baseline covariate and $p_1 = 10$ additional covariates, $miss_0 = 0$ and $miss_1 = 25\%$ missing values in baseline and additional covariates, respectively, missing at random (MAR) missingness, balanced outcome class distribution ($frac = 0.5$) and uncorrelated covariates ($\rho_1 = 0$). Results are shown for absence (theoretical $\Delta auc = 0$; **A,C,E,G**) and presence (theoretical $\Delta auc = 0.08$; **B,D,F,G**) of a moderate true added effect of the covariates on the outcome.

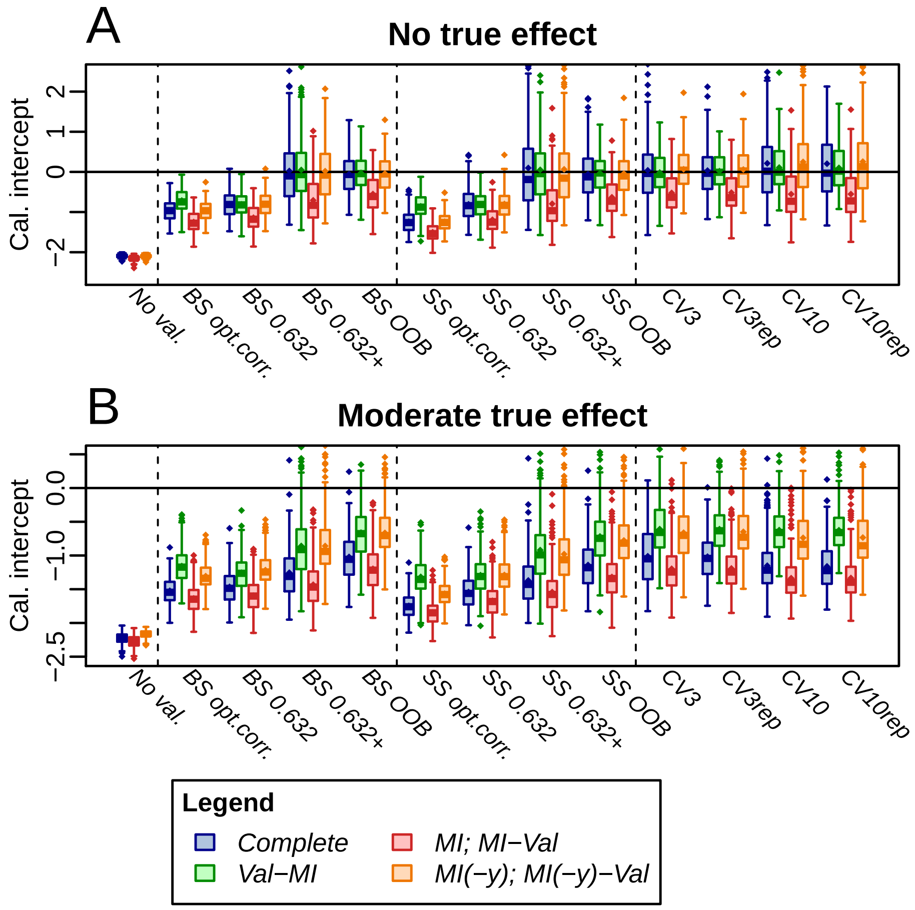

Figure S22: Simulation distribution of calibration intercept estimates obtained by different strategies. Boxplots showing distribution of calibration intercept estimates across the 250 simulated data sets in a setting with moderate sample size ($n = 200$), $p = 10$ covariates, moderate missing at random (MAR) missingness ($miss = 25\%$ of values missing), balanced outcome class distribution ($frac = 0.5$) and uncorrelated covariates ($\rho = 0$) in the absence (theoretical $auc = 0.5$; **A**) and presence (theoretical $auc = 0.66$; **B**) of a moderate true effect of the covariates on the outcome. The horizontal line denotes 'true' calibration intercept related to a complete data set of size 200 (see text). *BS*, bootstrap; *CVK(rep)*, (repeated) K-fold CV; *MI*, multiple imputation; *MI(-y)*, multiple imputation without including the outcome; *No val.*, no validation (i.e., apparent performance); *OOB*, out-of-bag estimate; *opt.corr.*, ordinary optimism-corrected estimate; *SS*, subsampling; *Val*, validation.

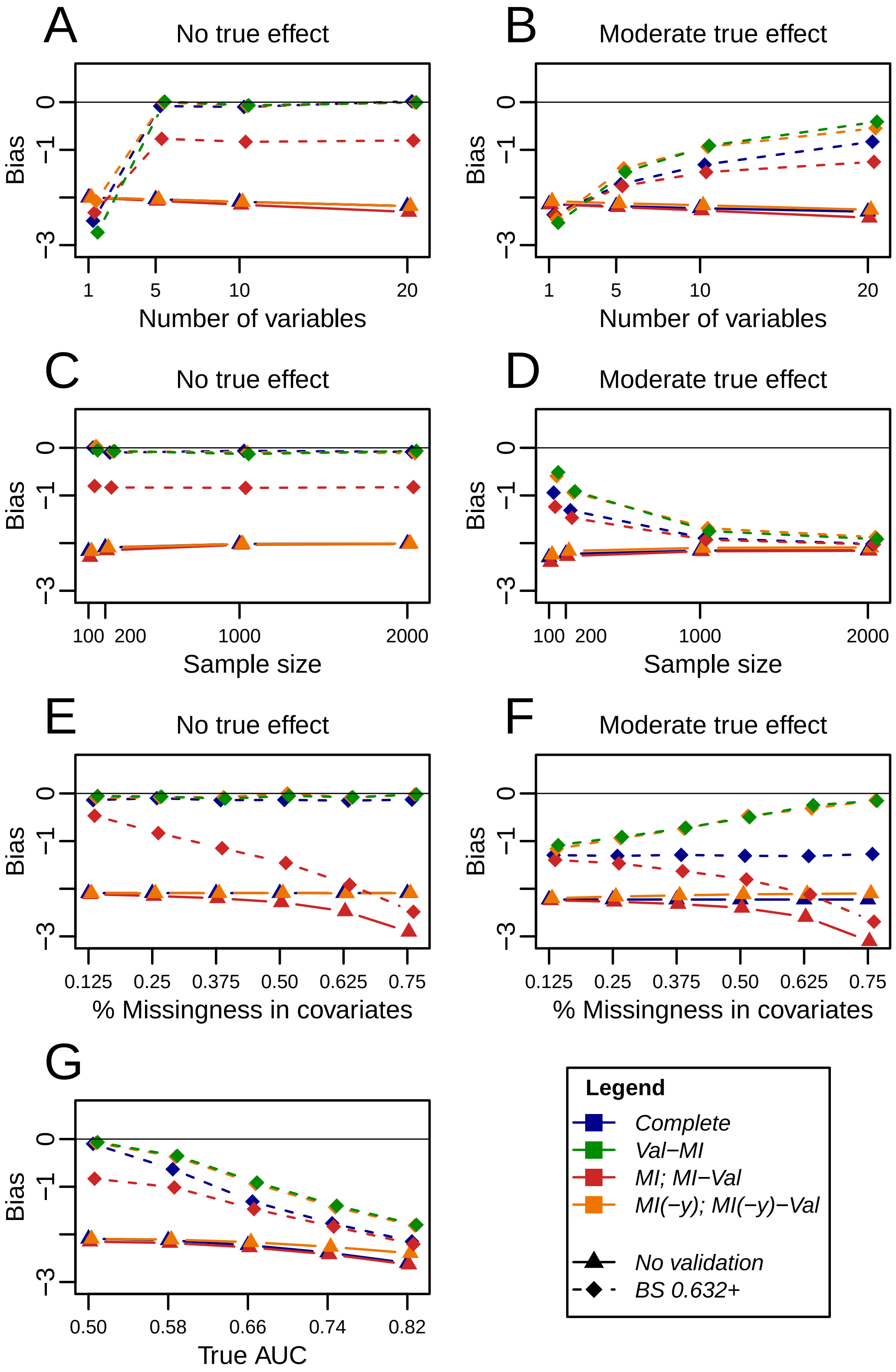

Figure S23: Bias of calibration intercept estimates obtained by different strategies based on bootstrapping. Bias is shown for one varying data set characteristic in each panel (**A,B** number of covariates p; **C,D** sample size n; **E,F** degree of missingness $miss$; **G** true effect auc), while keeping all remaining characteristics constant: sample size ($n = 200$), $p = 10$ covariates, 25% missing values, missing at random (MAR) missingness, balanced outcome class distribution ($frac = 0.5$), uncorrelated covariates ($\rho = 0$). Results are shown for absence (theoretical $auc = 0.5$; **A,C,E,G**) and presence (theoretical $auc = 0.66$; **B,D,F,G**) of a moderate true effect of the covariates on the outcome.

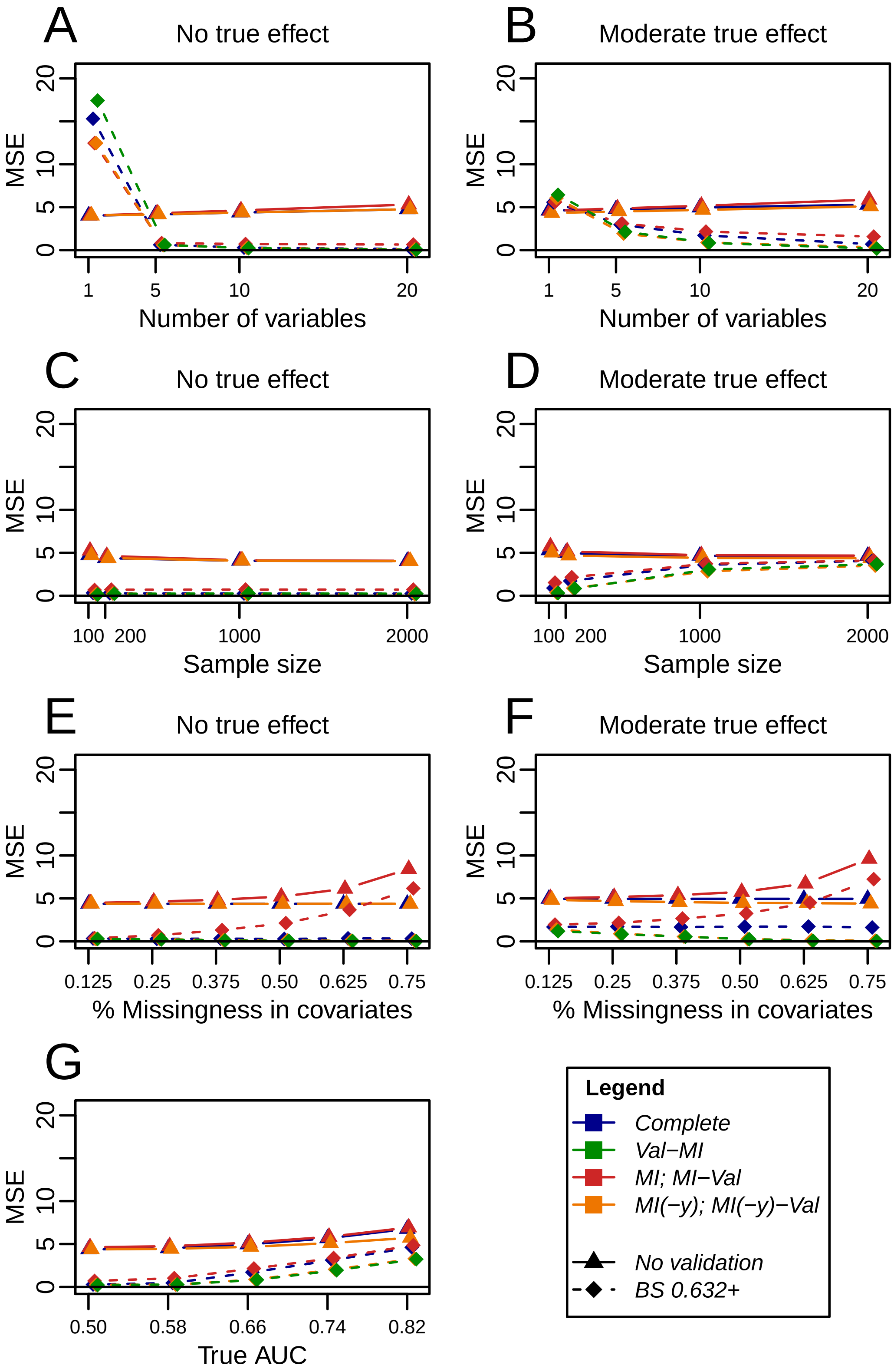

Figure S24: Mean squared error of calibration intercept estimates obtained by different strategies based on bootstrapping. Mean squared error (MSE) is shown for one varying data set characteristic in each panel (**A,B** number of covariates p; **C,D** sample size n; **E,F** degree of missingness $miss$; **G** true effect auc), while keeping all remaining characteristics constant: sample size ($n = 200$), $p = 10$ covariates, 25% missing values, missing at random (MAR) missingness, balanced outcome class distribution ($frac = 0.5$), uncorrelated covariates ($\rho = 0$). Results are shown for absence (theoretical $auc = 0.5$; **A,C,E,G**) and presence (theoretical $auc = 0.66$; **B,D,F,G**) of a moderate true effect of the covariates on the outcome.

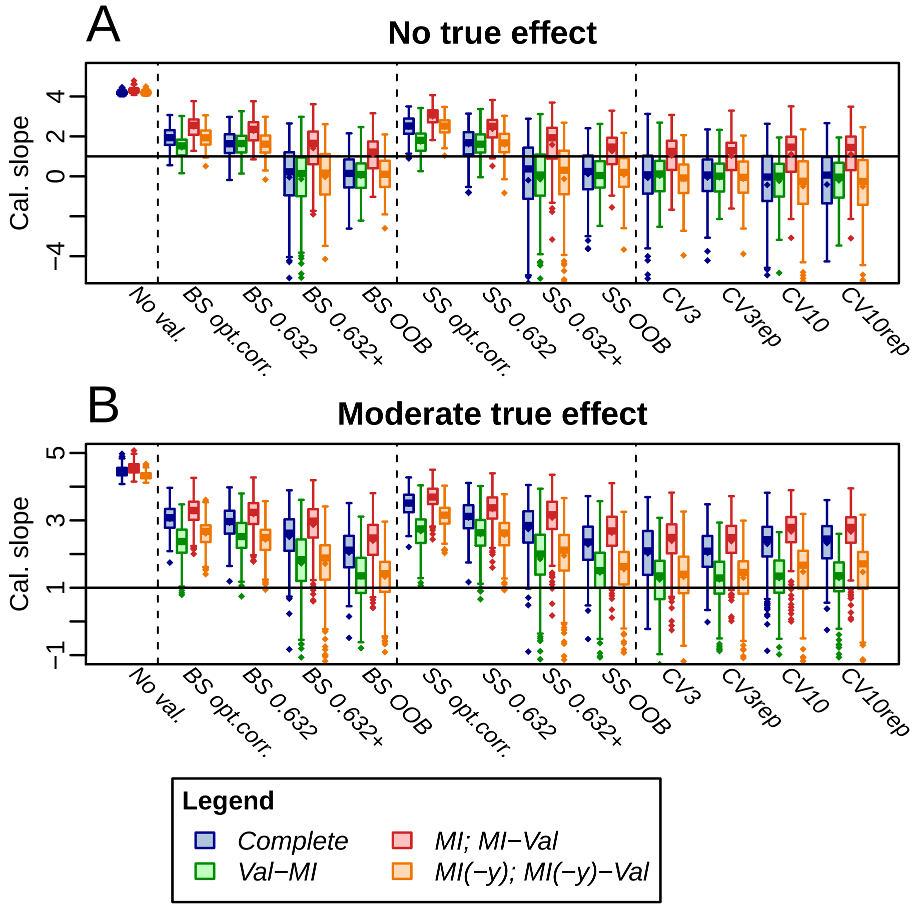

Figure S25: Simulation distribution of calibration slope estimates obtained by different strategies. Boxplots showing distribution of calibration slope estimates across the 250 simulated data sets in a setting with moderate sample size ($n = 200$), $p = 10$ covariates, moderate missing at random (MAR) missingness ($miss = 25\%$ of values missing), balanced outcome class distribution ($frac = 0.5$) and uncorrelated covariates ($\rho = 0$) in the absence (theoretical $auc = 0.5$; **A**) and presence (theoretical $auc = 0.66$; **B**) of a moderate true effect of the covariates on the outcome. The horizontal line denotes 'true' calibration slope related to a complete data set of size 200). *BS*, bootstrap; *CVK(rep)*, (repeated) K-fold CV; *MI*, multiple imputation; *MI(-y)*, multiple imputation without including the outcome; *No val.*, no validation (i.e., apparent performance); *OOB*, out-of-bag estimate; *opt.corr.*, ordinary optimism-corrected estimate; *SS*, subsampling; *Val*, validation.

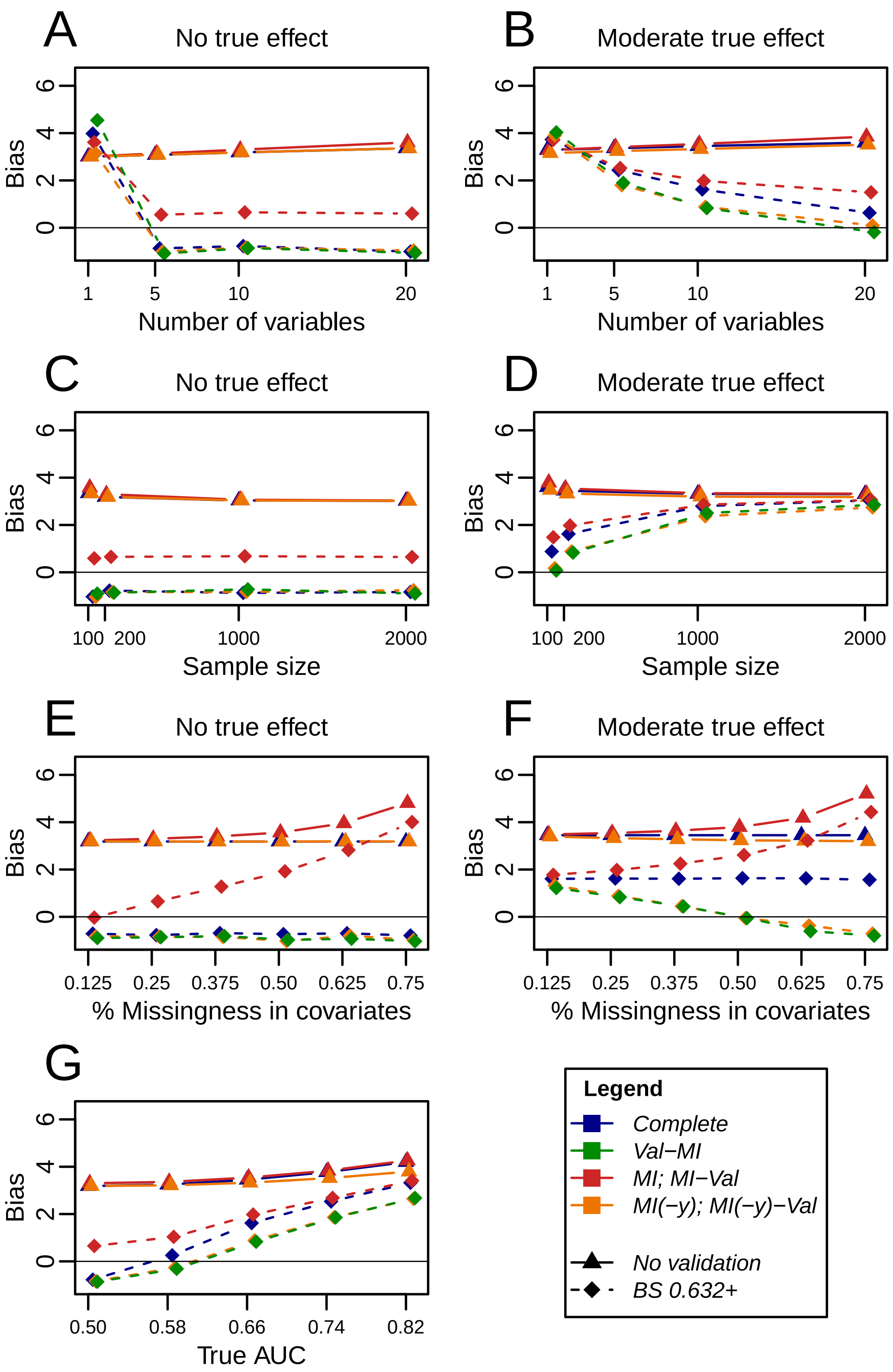

Figure S26: Bias of calibration slope estimates obtained by different strategies based on bootstrapping. Bias is shown for one varying data set characteristic in each panel (**A,B** number of covariates p; **C,D** sample size n; **E,F** degree of missingness $miss$; **G** true effect auc), while keeping all remaining characteristics constant: sample size ($n = 200$), $p = 10$ covariates, 25% missing values, missing at random (MAR) missingness, balanced outcome class distribution ($frac = 0.5$), uncorrelated covariates ($\rho = 0$). Results are shown for absence (theoretical $auc = 0.5$; **A,C,E,G**) and presence (theoretical $auc = 0.66$; **B,D,F,G**) of a moderate true effect of the covariates on the outcome.

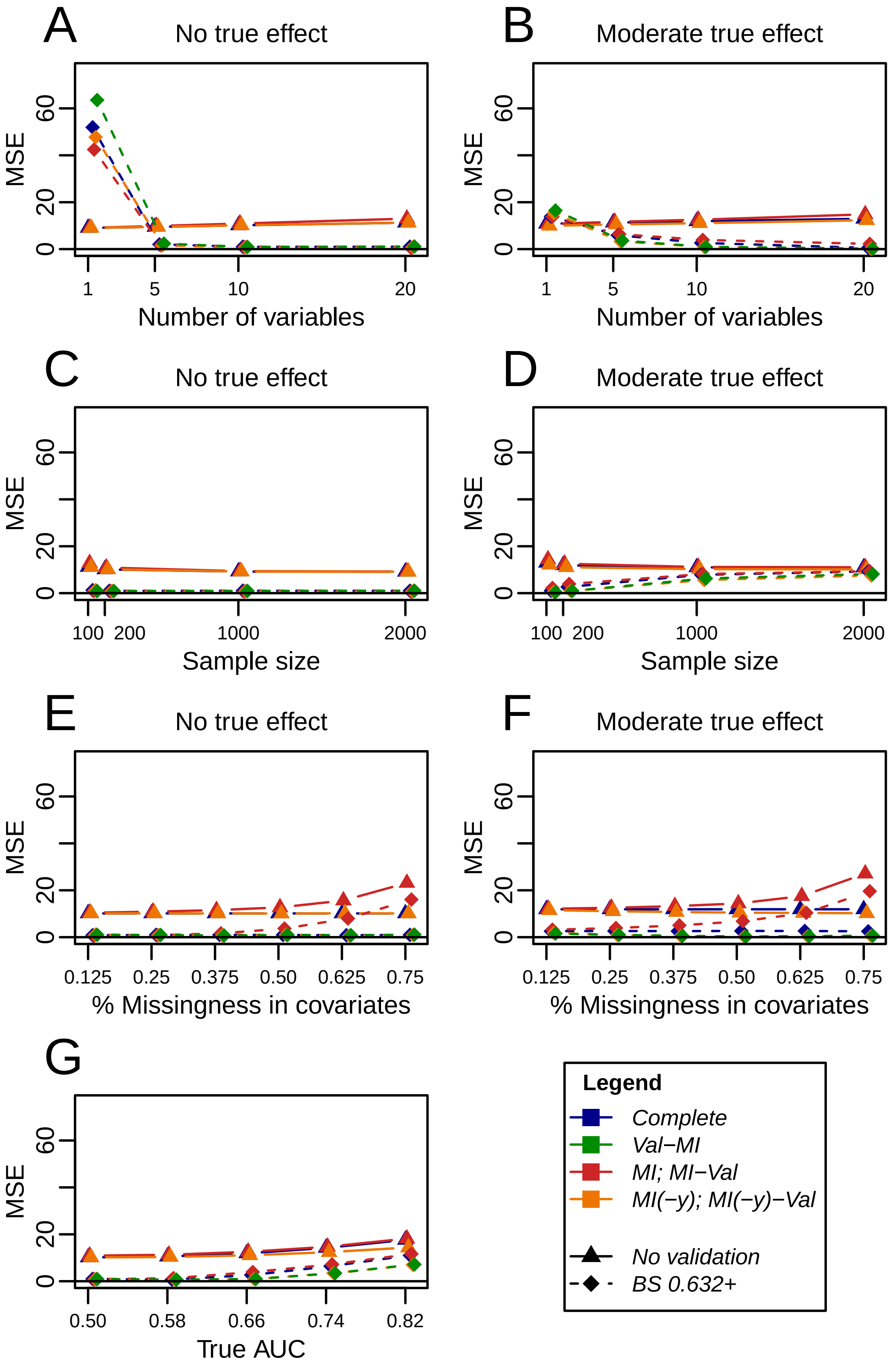

Figure S27: Mean squared error of calibration slope estimates obtained by different strategies based on bootstrapping. Mean squared error (MSE) is shown for one varying data set characteristic in each panel (**A,B** number of covariates p; **C,D** sample size n; **E,F** degree of missingness $miss$; **G** true effect auc), while keeping all remaining characteristics constant: sample size ($n = 200$), $p = 10$ covariates, 25% missing values, missing at random (MAR) missingness, balanced outcome class distribution ($frac = 0.5$), uncorrelated covariates ($\rho = 0$). Results are shown for absence (theoretical $auc = 0.5$; **A,C,E,G**) and presence (theoretical $auc = 0.66$; **B,D,F,G**) of a moderate true effect of the covariates on the outcome.

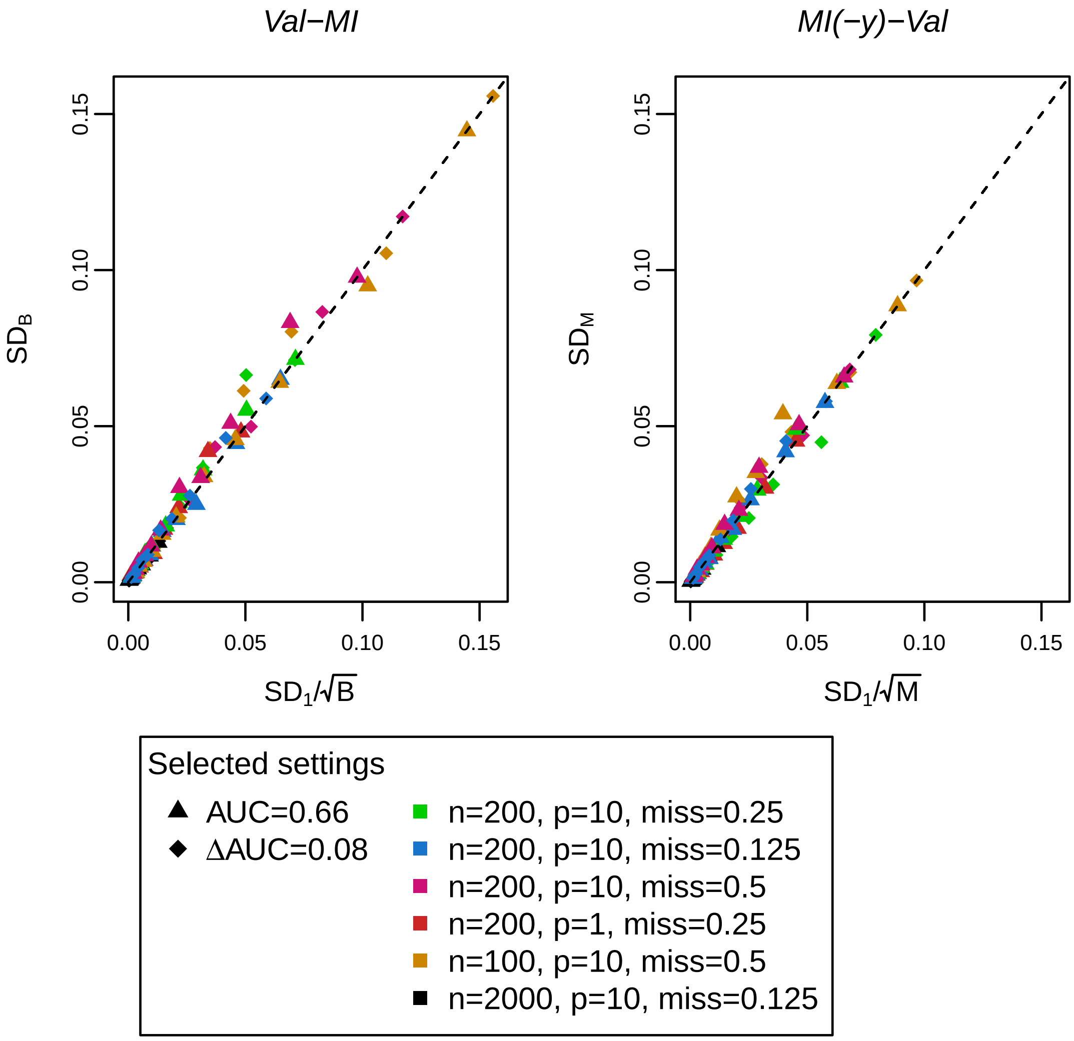

Figure S28: Relationship of standard deviations of performance estimates with varying number of bootstrap samples and imputations. Empirical confirmation of the analytical relationship between standard deviation (SD) of AUC or ΔAUC estimate with B as compared to 1 resamples and M as compared to 1 imputations for the strategies *Val-MI* and *MI(-y)-Val*, respectively, based on bootstrapping. Standard deviation were computed across 10 runs, and averaged across 10 simulated data sets. See Supplementary Figure S29 for a plot of SD's against B and M, respectively. Apart from the parameters provided in the legend, parameters were chosen as in Figure 3 in the main manuscript. In the case of added performance, the number of variables in the baseline model was set to $p_0 = 1$. MI, multiple imputation; Val, internal.

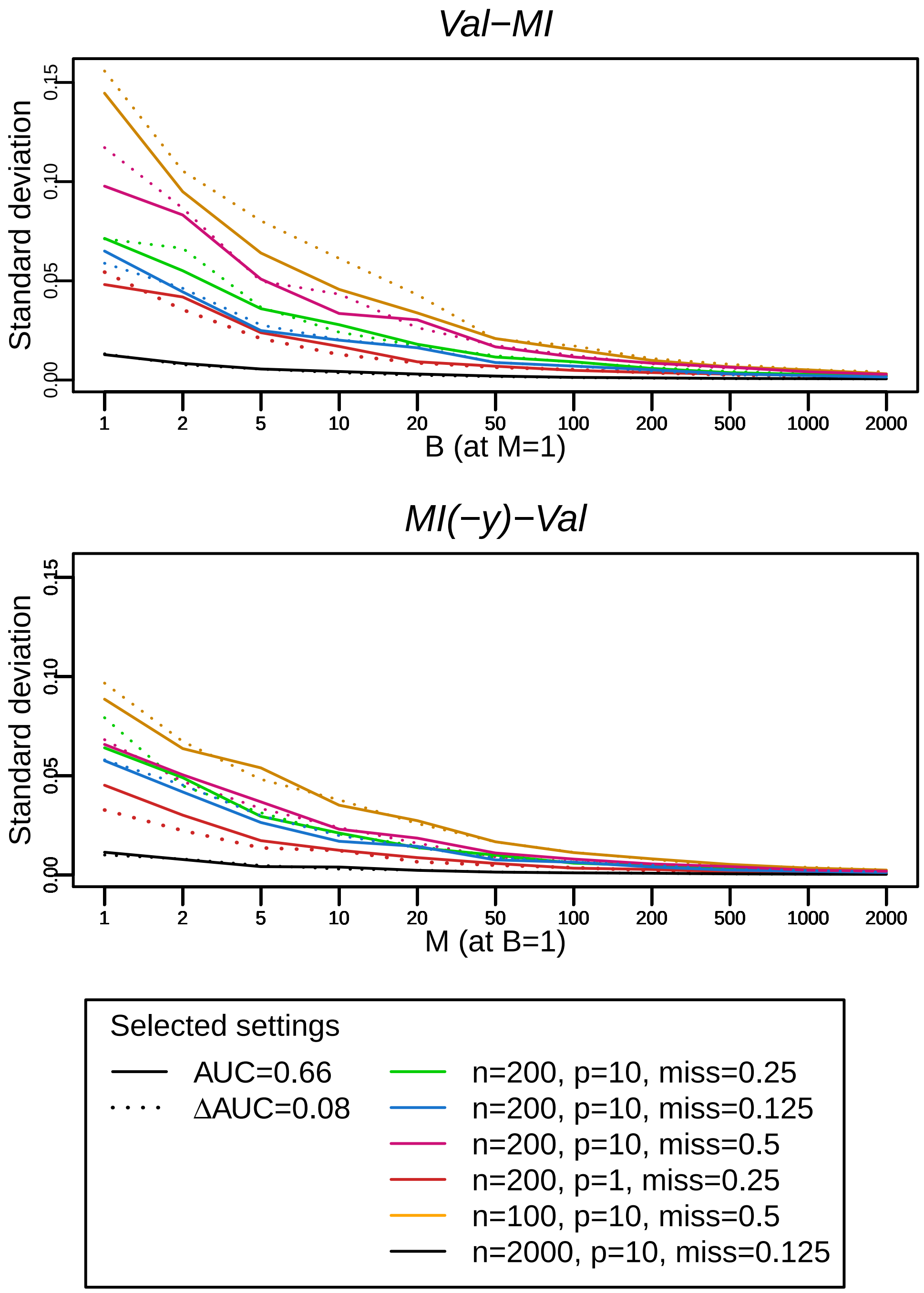

Figure S29: Standard deviation of performance estimates at varying number of resamples and imputations. Shown for AUC and ΔAUC for strategies *Val-MI* and *MI(-y)-Val* based on bootstrapping at varying number of bootstrap samples B and number of imputations M. Standard deviations were computed across 10 runs, and averaged across 10 simulated data sets. Apart from the parameters provided in the legend, parameters were chosen as in Figure 3 in the main manuscript. In the case of added performance, the number of variables in the baseline model was set to $p_0 = 1$. MI, multiple imputation; Val, validation.

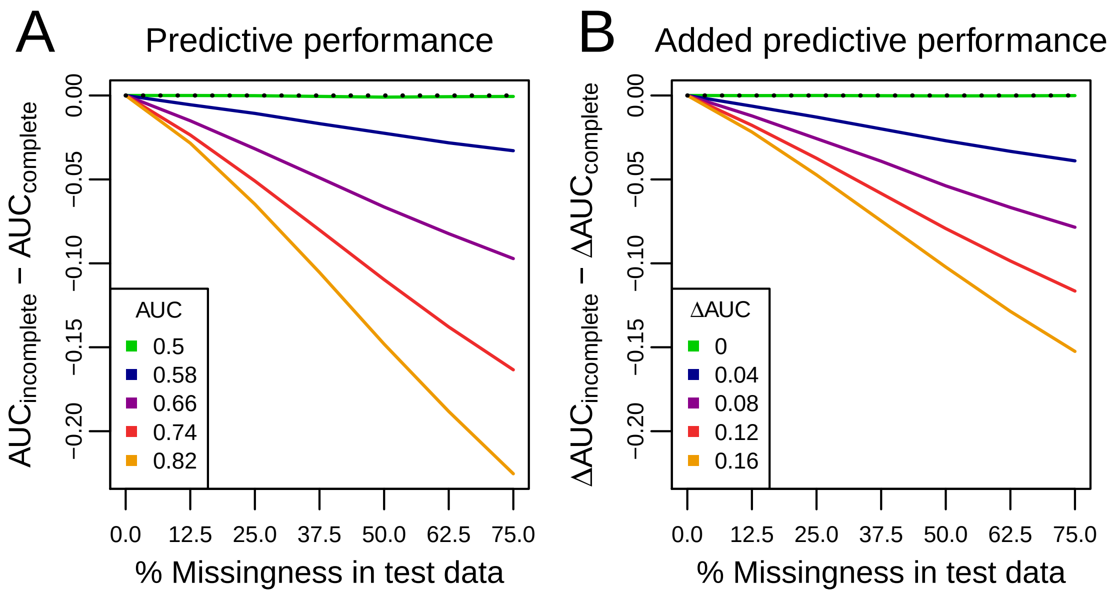

Figure S30: Impairment of performance evaluation through missing values in the test data. Deviation of estimated AUC (**A**) and ΔAUC (**B**) when the model was fitted to complete data ($n = 200$; theoretical performance as indicated in the legends) and evaluated in incomplete as compared to complete large test data ($n = 10,000$, same underlying simulated effect sizes). Parameters were chosen as follows: $p = 10$ (**A**) and $p_0 = 1$,$p_1 = 10$ (**B**), and otherwise as in Figure 3 in the main manuscript, and averaged across 250 simulations is shown.

Table S1: Descriptive information of phenotypic and inflammation-related markers from the MONICA/KORA subcohort.

Variable (categories)	Counts	Frequencies (%)	Missing values (%)
Sex (male/female)	1059/953	52.6/47.4	0.00
Survey (S1/S2/S3)	789/737/486	39.2/36.6/24.2	0.00
Smoking status (current/former/never)	522/561/928	26.0/27.9/46.1	0.05
Physical activity (inactive/active)	1246/763	62.0/38.0	0.15
**Variable (unit)**	**Mean (SD)**	**Range**	**Missing values (%)**
Age (years)	51.7 (10.2)	34.0, 75.0	0.00
SBP (mmHg)	133.1 (18.6)	92.0, 227.0	0.00
TC/HDL ratio	4.5 (1.7)	1.5, 26.4	0.05
Alcohol (g/d)	21.9 (27.7)	0.0, 244.6	0.20
BMI (kg/m2)	27.0 (4.0)	16.6, 49.3	0.35
CRP (mg/l)	3.0 (6.0)	0.0, 90.8	0.25
E-Selectin (ng/ml)	56.2 (28.0)	11.8, 446.6	1.54
ICAM (ng/ml)	770.2 (292.4)	80.5, 3200.0	1.54
IL-6 (pg/ml)	3.4 (8.2)	0.1, 176.3	1.79
IL-18 (pg/ml)	204.9 (240.6)	4.9, 8792.0	2.14
MCP-1 (pg/ml)	235.5 (152.4)	2.4, 1340.0	2.14
IP-10 (pg/ml)	295.0 (285.5)	9.8, 5100.0	2.19
IL-8 (pg/ml)	10.4 (22.1)	0.3, 645.7	2.24
RANTES (pg/ml)	29000.0 (16793.4)	3743.0, 176000.0	5.62
MIF (ng/ml)	19.3 (9.3)	4.5, 165.4	5.72
Leptin (ng/ml)	14.3 (15.2)	0.2, 121.0	14.02
MPO (mg/ml)	137.3 (71.5)	23.1, 1226.0	14.12
Adiponectin (ug/ml)	12.0 (4.4)	2.3, 29.2	14.26
TGF-beta1 (ng/ml)	35.5 (7.5)	6.1, 60.6	14.26
25(OH)D (nmol/l)	45.0 (19.9)	5.1, 153.9	26.44

25(OH)D, 25-hydroxy-vitamin D; CRP, C-reactive protein; HDL, high density lipoprotein (cholesterol); ICAM, intercellular adhesion molecule; IL-6,8,18; interleukin 6,8,18; IP-10, interferon γ-induced protein 10; MCP-1, monocyte chemotactic protein 1; MIF, macrophage migration inhibitory factor; MPO, myeloperoxidase; RANTES, regulated on activation, normal T cell expressed and secreted; SBP, systolic blood pressure; TC, total cholesterol; TGF-beta1, transforming growth factor β1.

Table S2: Standard deviation of performance estimates obtained with *Val-MI* based on bootstrapping at one resample ($B = 1$) and one imputation ($M = 1$). Shown for AUC (where $p_0 = 0$) and ΔAUC (where $p_0 > 0$) for varying parameter combinations (as indicated in the table; otherwise chosen as in Figure 3 in the main manuscript). Standard deviations were computed across 50 runs, averaged across 10 simulated data sets for each parameter combination. MI, multiple imputation; Val, validation.

		**Sample size (n)**	100				200				1000			
		**Number of covariates (p)**	1	5	10	20	1	5	10	20	1	5	10	20
**Number of baseline covariates (p_0)**	**Proportion of missing values**													
0	0.125		0.0678	0.0855	0.1005	0.1052	0.0467	0.0577	0.0652	0.0720	0.0168	0.0201	0.0197	0.0224
	0.25		0.0810	0.1034	0.1088	0.1088	0.0569	0.0697	0.0795	0.0862	0.0207	0.0225	0.0232	0.0282
	0.375		0.1134	0.1211	0.1357	0.1164	0.0746	0.0911	0.0939	0.0999	0.0266	0.0291	0.0320	0.0369
	0.5		0.1388	0.1394	0.1403	0.1226	0.0954	0.1007	0.1200	0.1123	0.0314	0.0365	0.0401	0.0520
	0.625		0.1356	0.1456	0.1469	0.1144	0.1066	0.1259	0.1345	0.1091	0.0382	0.0484	0.0575	0.0668
	0.75		0.1485	0.1372	0.1253	0.1124	0.1359	0.1440	0.1350	0.1045	0.0526	0.0707	0.0818	0.0888
1	0.125		0.0733	0.0948	0.1134	0.1194	0.0436	0.0593	0.0683	0.0841	0.0189	0.0190	0.0194	0.0224
	0.25		0.0821	0.1086	0.1283	0.1356	0.0469	0.0701	0.0848	0.0947	0.0250	0.0205	0.0237	0.0277
	0.375		0.0983	0.1287	0.1453	0.1367	0.0562	0.0793	0.0938	0.1128	0.0348	0.0263	0.0293	0.0356
	0.5		0.1069	0.1486	0.1568	0.1405	0.0735	0.1012	0.1195	0.1140	0.0433	0.0340	0.0395	0.0520
	0.625		0.1212	0.1557	0.1573	0.1494	0.0849	0.1241	0.1305	0.1124	0.0484	0.0445	0.0544	0.0663
	0.75		0.1176	0.1478	0.1427	0.1351	0.0985	0.1449	0.1435	0.1145	0.0738	0.0668	0.0842	0.0884
10	0.125		0.0528	0.0841	0.1018	0.1272	0.0408	0.0531	0.0637	0.0723	0.0173	0.0188	0.0210	0.0219
	0.25		0.0672	0.0926	0.1200	0.1378	0.0481	0.0617	0.0730	0.0931	0.0196	0.0222	0.0233	0.0290
	0.375		0.0705	0.1194	0.1340	0.1410	0.0557	0.0787	0.0948	0.1052	0.0242	0.0265	0.0294	0.0308
	0.5		0.0889	0.1309	0.1367	0.1382	0.0604	0.0983	0.1151	0.1033	0.0282	0.0330	0.0399	0.0471
	0.625		0.0981	0.1389	0.1311	0.1340	0.0805	0.1124	0.1246	0.1083	0.0325	0.0439	0.0564	0.0662
	0.75		0.0934	0.1265	0.1264	0.1230	0.0804	0.1273	0.1183	0.1070	0.0387	0.0637	0.0765	0.0882

Table S3: Standard deviation of performance estimates obtained with *MI(-y)-Val* based on bootstrapping at one imputation ($M = 1$) and one resample ($B = 1$). Shown for AUC (where $p_0 = 0$) and ΔAUC (where $p_0 > 0$) for varying parameter combinations (as indicated in the table; otherwise chosen as in Figure 3 in the main manuscript). Standard deviations were computed across 50 runs, averaged across 10 simulated data sets for each parameter combination. MI, multiple imputation; Val, validation.

		Sample size (n)	100				200				1000			
		Number of covariates (p)	1	5	10	20	1	5	10	20	1	5	10	20
Number of baseline covariates (p_0)	Proportion of missing values													
0	0.125		0.0645	0.0704	0.0886	0.0973	0.0427	0.0559	0.0593	0.0686	0.0159	0.0184	0.0168	0.0209
	0.25		0.0739	0.0829	0.0953	0.1044	0.0432	0.0572	0.0605	0.0680	0.0168	0.0188	0.0195	0.0225
	0.375		0.0825	0.0938	0.0974	0.0974	0.0536	0.0605	0.0670	0.0706	0.0198	0.0215	0.0216	0.0270
	0.5		0.0956	0.0975	0.0994	0.1092	0.0643	0.0634	0.0673	0.0712	0.0191	0.0245	0.0267	0.0314
	0.625		0.0952	0.0969	0.1031	0.1037	0.0625	0.0676	0.0679	0.0740	0.0233	0.0255	0.0304	0.0334
	0.75		0.0933	0.0950	0.1048	0.1013	0.0660	0.0674	0.0699	0.0740	0.0317	0.0295	0.0321	0.0331
1	0.125		0.0720	0.0857	0.1020	0.1139	0.0346	0.0538	0.0637	0.0758	0.0157	0.0167	0.0181	0.0190
	0.25		0.0735	0.0936	0.1074	0.1234	0.0321	0.0587	0.0623	0.0764	0.0156	0.0174	0.0188	0.0218
	0.375		0.0658	0.1000	0.1168	0.1240	0.0377	0.0625	0.0641	0.0764	0.0151	0.0164	0.0189	0.0219
	0.5		0.0643	0.0930	0.1092	0.1249	0.0347	0.0616	0.0680	0.0794	0.0136	0.0162	0.0180	0.0233
	0.625		0.0669	0.1028	0.1112	0.1237	0.0304	0.0626	0.0683	0.0836	0.0113	0.0160	0.0188	0.0228
	0.75		0.0708	0.0967	0.1157	0.1249	0.0314	0.0597	0.0708	0.0806	0.0087	0.0147	0.0169	0.0247
10	0.125		0.0462	0.0698	0.0857	0.1169	0.0360	0.0492	0.0550	0.0672	0.0163	0.0165	0.0193	0.0196
	0.25		0.0469	0.0680	0.0873	0.1143	0.0366	0.0497	0.0519	0.0688	0.0137	0.0169	0.0182	0.0201
	0.375		0.0442	0.0724	0.0940	0.1154	0.0346	0.0492	0.0541	0.0663	0.0129	0.0174	0.0188	0.0217
	0.5		0.0448	0.0741	0.0921	0.1234	0.0307	0.0498	0.0575	0.0743	0.0110	0.0164	0.0196	0.0212
	0.625		0.0387	0.0690	0.0886	0.1109	0.0323	0.0480	0.0559	0.0744	0.0102	0.0158	0.0192	0.0228
	0.75		0.0388	0.0717	0.0903	0.1118	0.0259	0.0466	0.0554	0.0682	0.0084	0.0143	0.0191	0.0225
